# Communication skills training for improving the communicative abilities of student social workers

**DOI:** 10.1002/cl2.1309

**Published:** 2023-02-23

**Authors:** Emma Reith‐Hall, Paul Montgomery

**Affiliations:** ^1^ Health and Life Sciences De Montfort University Leicester UK; ^2^ Department of Social Policy and Social Work University of Birmingham Birmingham UK

## Abstract

**Background:**

Good communication is central to effective social work practice, helping to develop constructive working relationships and improve the outcomes of people in receipt of social work services. There is strong consensus that the teaching and learning of communication skills for social work students is an essential component of social work qualifying courses. However, the variation in communication skills training and its components is significant. There is a sizeable body of evidence relating to communication skills training therefore a review of the findings helps to clarify what we know about this important topic in social work education. We conducted this systematic review to determine whether communication skills training for social work students works and which types of communication skills training, if any, were more effective and lead to the most positive outcomes.

**Objectives:**

This systematic review aimed to critically evaluate all studies which have investigated the effectiveness of communication skills training programmes for social work students. The research question which the review posed is: ‘What is the effectiveness of communication skills training for improving the communicative abilities of social work students?’ It was intended that the review would provide a robust evaluation of communication skills training for social work students and help explain variations in practice to support educators and policy‐makers to make evidence‐based decisions in social work education, practice and policy.

**Search Methods:**

We conducted a search for published and unpublished studies using a comprehensive search strategy that included multiple electronic databases, research registers, grey literature sources, and reference lists of prior reviews and relevant studies.

**Selection Criteria:**

Study selection was based on the following characteristics: Participants were social work students on generic (as opposed to client specific) qualifying courses; Interventions included any form of communication skills training; eligible studies were required to have an appropriate comparator such as no intervention or an alternative intervention; and outcomes included changes in knowledge, attitudes, skills and behaviours. Study selection was not restricted by geography, language, publication date or publication type.

**Data Collection and Analysis:**

The search strategy was developed using the terms featuring in existing knowledge and practice reviews and in consultation with social work researchers, academics and the review advisory panel, to ensure that a broad range of terminology was included. One reviewer conducted the database searches, removing duplicates and irrelevant records, after which each record was screened by title and abstract by both reviewers to ensure robustness. Any studies deemed to be potentially eligible were retrieved in full text and screened by both reviewers.

**Main Results:**

Fifteen studies met the inclusion criteria. Overall, findings indicate that communication skills training including empathy can be learnt, and that the systematic training of social work students results in some identifiable improvements in their communication skills. However, the evidence is dated, methodological rigour is weak, risk of bias is moderate to high/serious or incomplete, and extreme heterogeneity exists between the primary studies and the interventions they evaluated. As a result, data from the included studies were incomplete, inconsistent, and lacked validity, limiting the findings of this review, whilst identifying that further research is required.

**Authors’ Conclusions:**

This review aimed to examine effects of communication skills training on a range of outcomes in social work education. With the exception of skill acquisition, there was insufficient evidence available to offer firm conclusions on other outcomes. For social work educators, our understanding of how communication skills and empathy are taught and learnt remain limited, due to a lack of empirical research and comprehensive discussion. Despite the limitations and variations in educational culture, the findings are still useful, and suggest that communication skills training is likely to be beneficial. One important implication for practice appears to be that the teaching and learning of communication skills in social work education should provide opportunities for students to practice skills in a simulated (or real) environment. For researchers, it is clear that further rigorous research is required. This should include using validated research measures, using research designs which include appropriate counterfactuals, alongside more careful and consistent reporting. The development of the theoretical underpinnings of the interventions used for the teaching and learning of communication skills in social work education is another area that researchers should address.

## 1 PLAIN LANGUAGE SUMMARY

### 1.1 Communication skills training helps improve how social work students interact with the people they safeguard and support

Communication skills training, including empathy training, can help social work students to develop their communication skills. Opportunities to practise communication skills in a safe and supportive environment through role‐play and/or simulation, with feedback and reflection, helps students to improve their skills. The effect of doing this training face‐to‐face, online or via blended learning is largely unknown.

### 1.2 What is this review about?

Good communication skills are important for social work practice and are commonly taught on social work qualifying courses. There is a range of different types of educational interventions, with wide variations in theoretical basis, approach, duration and mode of delivery. This systematic review looks at whether different interventions are effective in producing the following outcomes: social work students’ knowledge, attitudes, skills and behaviours.

**What is the aim of this review?**

This Campbell systematic review assesses whether communication skills training for social work students works, and which types of communication skills training, if any, were more effective and led to the most positive outcomes.

### 1.3 What studies are included?

This review summarises quantitative data from randomised and non‐randomised studies. The 15 studies included in this review were undertaken in Canada, Australia and North America. The research is very limited in terms of scope and quality, and there are important weaknesses in the evidence base.

### 1.4 Does communication skills training improve the communicative abilities of social work students?

Systematic communication skills training shows some promising effects in the development of social work students’ communicative abilities, especially in terms of their ability to demonstrate empathy and interviewing skills.

### 1.5 What do the findings of this review mean?

Communication is very important for social work practice, so we need to ensure that student social workers have opportunities to develop their communication skills.

Too few studies fully assessed student characteristics such as age, sex and ethnicity or took account of how previous experience, commitments and motivation affected students’ learning.

Consideration of stakeholder involvement and collaboration (such as by people with lived experience) was also lacking. Only the role of the educator was considered.

The studies were largely of poor quality and investigated many different implementation features, which made it difficult to draw any firm conclusions about what makes the teaching and learning of communication skills in social work education effective.

Researchers conducting studies into communication skills training should seek to carry out robust and rigorous outcomes‐focused studies. They should also consider trying to see how and where these interventions might work, as well as understanding for whom they may be effective.

### 1.6 How up‐to‐date is this review?

The review authors searched for studies that had been published until 15 June 2021.

## 2 BACKGROUND

### 2.1 Description of the condition

2.1.1

Good communication is central to social work practice (Koprowska, 2020; Lishman, 2009), underpinning the success of a wide range of social work activities. People in receipt of social work services value social workers who are warm, empathic, respectful, good at listening and demonstrate understanding and compassion (Beresford et al., 2008; Department of Health, 2002; Ingram, 2013; Kam, 2020; Munford & Sanders, 2015; Social Care Institute for Excellence, 2000; Tanner, 2019). Even in diverse and challenging circumstances, effective communication is thought to build constructive working relationships and enhance social work outcomes (Healy, 2018).

Communication, sometimes referred to as interpersonal communication, ‘involves two (or more) people interacting to exchange information and views’ (Beesley et al., 2018). It is driven and directed by the desire to achieve particular goals and is underpinned by perceptual, cognitive, affective and behavioural operations (Hargie, 2017). In social work practice and education, the values of the profession and the specific social, cultural, political and ideological contexts in which social workers operate, influence the nature of interpersonal communication (Harms, 2015; Koprowska, 2020; Thompson, 2003).

Research has tended to focus on particular aspects of communication, or the impact of communication in specific contexts. For example, a study examining how social workers communicate with parents in child protection scenarios, identified that social workers who demonstrated empathy towards simulated clients, encountered less resistance and more disclosure (Forrester et al., 2008). Social workers who use creative and play‐based approaches confidently facilitate engagement and communication with children (Ferguson, 2016; Handley & Doyle, 2014). Adapting skills and strategies to address specific communication difficulties is equally important in social work with adults. Offering choices through pictographs can help people with Aphasia to answer open questions (Rowland & McDonald, 2009), whilst Talking Mats can facilitate conversation with people who have dementia (Murphy et al., 2007). Research into the experiences and preferences of palliative care patients found small impactful supererogatory acts demonstrated compassion which allowed them to ‘feel heard, understood, and validated’ (Sinclair et al., 2017, p. 446). Communicating effectively with adults in receipt of health and social care services also enables them to better participate in important decisions about their care.

The impact of failing to communicate effectively has been well documented, particularly through reports into incidents of child deaths (Laming, 2003, 2009; Munro, 2011). Consequently, the importance of teaching communication skills to social work students as a means of enabling them to communicate effectively has long been recognised (Smith, 2002). More recently, there have been calls for the expansion and/or improvement of this training (Luckock et al., 2006; Narey, 2014). Considerable time, effort and money has been spent on achieving this aim, leading to a wide range of communication skills training courses becoming embedded in social work programmes across the globe. Communication generally, and some communication skills specifically, feature in the educational standards of different countries including the Australian Social Work Education and Accreditation standards, the Educational Policy Accreditation Standards in the US and the Professional Capabilities Framework in the UK (Australian Association of Social Workers, 2020; British Association of Social Workers, 2018; Council on Social Work Education, 2015). One of the consequences of the coronavirus pandemic was increasing diversification of the delivery of teaching and learning in Higher Education. However, the impact of online or blended learning on the development of student social workers’ communication skills remains to be seen.

### 2.2 Description of the intervention

Communication skills training (CST) can be defined as ‘any form of structured didactic, e‐learning, and experiential (e.g., using simulation and role‐play) training used to develop communicative abilities’ (Papageorgiou et al., 2017, p. 6). Although ‘communication skills training’ (CST) is the name given to the intervention on a wide range of professional and vocational courses, in social work education, the intervention is more commonly referred to as the ‘teaching and learning of communication skills’; a trend reflected in the titles of various knowledge and practice reviews. Given purpose, role and context have a significant impact on communication in social work practice, conceptualisations which integrate knowledge, values and skills, for example the knowing, being and doing domains developed by Lefevre and colleagues (Lefevre et al., 2008) have become increasingly popular (Ayling, 2012; Woodcock Ross, 2016). In social work education, the intervention includes not only communication processes, but also an understanding of the broader contextual issues in which those interactions in social work practice occur. This views communication in social work as both an art and a science (Healy, 2018), which alongside a move away from purely instructional methods, helps explain the preference for the term ‘teaching’ or ‘education’ rather than ‘training’ among social work academics and researchers. There is a tendency within this discipline for significant variation in terminology due to the wide knowledge base from which social work draws. The term ‘communication skills’ is not applied uniformly in the social work literature‐microskills, interpersonal skills and interviewing skills are frequently used alternatives.

In spite of a lack of consensus about what the intervention is called, ‘the inclusion of a dedicated communication skills module early in the course, or a strong communication component within an early module about methods, skills and practice’ is commonplace (Dinham, 2006, p. 841). A consensus appears to be emerging in the wider social work literature, regarding what the basic communication skills for social work practice actually entail. These microskills comprise non‐verbal communication such as making eye contact and nodding, alongside a range of verbal techniques including clarifying, reflecting, paraphrasing, summarising and asking open questions. Described in detail in a number of social work text books (Beesley et al., 2018; Cournoyer, 2016; Healy, 2018; Sidell & Smiley, 2008), and featuring in the educational standards, competency and capability frameworks of various countries (Australian Association of Social Workers, 2020; British Association of Social Workers, 2018; Council on Social Work Education, 2015), these skills form part of the content of a number of communication skills courses and preparation for practice modules. Microskills help social workers and social work students to ‘establish and maintain empathy, communicate non‐verbally and verbally in effective ways, establish the context and purpose of the work, open an interview, actively listen, establish the story or the nature of the problem, ask questions, intervene and respond appropriately’ (Harms, 2015, p. 22). Microskills are considered to be transferable across client groups and settings.

Using case study scenarios that students might encounter in practice, microskills are rehearsed in different social work contexts or circumstances. Typically, students practice the microskills through undertaking simulated social work tasks such as assessments and care planning. When applied to social work tasks and contexts, communication skills are sometimes referred to within the social work literature as interviewing skills. An interview is a ‘person‐to‐person interaction that has a definite and deliberate purpose’ (Kadushin & Kadushin, 2013, p. 6). It is through social work interviews that ‘important connections and relationships are developed, and where important concepts such as partnership and empowerment are taken forward’ (Trevithick, 2012, p. 185).

The pedagogic practices used to teach communication skills to social work students include a wide range of affective, cognitive and behavioural components, whereby students participate in a variety of activities. Following face‐to‐face taught input including theory, communication skills are generally rehearsed, using role‐play with peers (e.g., Koprowska, 2003), simulated practice with service users (e.g., Moss et al., 2007) or actors (e.g., Petracchi & Collins, 2006). Tutors and peers may also model communication skills to demonstrate different techniques. Critical reflection, which facilitates students’ self‐awareness is encouraged. Feedback is an important component in helping learners develop an understanding of their strengths and areas for development, and a range of feedback mechanisms are welcomed by students (Tompsett, Henderson, Mathew Byrne, et al., 2017). Video and playback are often used to support the learning that occurs through feedback and reflective processes. Some universities have purpose‐built recording suites or provide students with equipment such as tablets to facilitate the recording of communication skills practice. The rationale for video and playback is ‘that each student's adult ability to be their own best assessor’ is ‘utilised to the full’ (Moss et al., 2007, p. 715); the value of which has been recognised by students elsewhere (Bolger, 2014; Cartney, 2006). A learning environment, characterised by trust, safety and security, appears to be an important mechanism for students to make use of experiential activities. Opportunities for observing skills in practice, through shadowing a social worker or allied practitioner, feature in some communication skills or preparation for practice modules. Attention may also be devoted to specific areas of communication: communicating with children, communicating with people who have hearing impairments, and inter‐professional communication are some examples.

No specific blueprint for CST in social work exists, thus the nature of the training sessions and course length vary from one educational institution to another. Typically, in the UK, CST is delivered to first year undergraduate and postgraduate students before they commence their first practice placement: in England, this may comprise some of the 30 days of skills training which universities typically provide. Content and teaching activities tend to be designed and delivered on an individual basis by social work academics, often with involvement from people with lived experience (service users and carers), practitioners and local employers. Examples of gap‐mending strategies for user involvement are beginning to find their way into the literature (Askheim et al., 2017) and have been applied to the teaching and learning of communication skills (Reith‐Hall, 2020), however such activities are far from mainstream. Minimum requirements, dosage, and delivery methods are not prescribed, leading to considerable heterogeneity of the intervention in practice.

### 2.3 How the intervention might work

Training or education‐based interventions aimed at improving the communicative abilities of student social workers seek to bring about changes in learners’ knowledge, values and skills in terms of how to communicate effectively in social work practice.

Psychological perspectives and counselling theories, including the work of humanistic and client‐centred theorists such as Rogers, Carkhuff and Egan tend to underpin microskills training. Other communication theories, including the model of interpersonal communication developed by Hargie (Hargie, 2006) also provide a theoretical basis for the skills taught on some of these courses. Concerns have been raised that psychological and counselling theories have been applied to social work uncritically (Trevithick et al., 2004), without due consideration of the challenges this may present. A number of social work academics have pulled together theory and research on communication skills in recent years (e.g., Beesley et al., 2018; Harms, 2015; Healy, 2018; Koprowska, 2020; Lishman, 2009; Woodcock Ross, 2016) in an attempt to address this issue. Nonetheless, it still remains ‘difficult to identify a coherent theoretical framework that informs the learning and teaching of communication skills in social work’ (Trevithick et al., 2004, p. 18).

The theoretical underpinnings of the pedagogic practices used to teach communication skills are not always clear (Dinham, 2006; Trevithick et al., 2004). The conception of reflection in and on action (Schön, 1983) and the importance of ‘learning by doing’ (Schön, 1987, p. 17) are often cited as underpinning the teaching of communication skills modules in social work education. Experiential learning, ‘the process whereby knowledge is created through the transformation of experience’ (Kolb, 1984, p. 38) is another of the prevailing philosophies, although Trevithick et al., 2004, p. 24) suggest there is an uncritical assumption that ‘experiential is best’. Reference is sometimes made to theories of adult learning, whereby students are expected to draw on their own experiences, take responsibility for their own learning, and engage in peer learning. This mode of learning ‘is understood to encourage the sustained internalisation of skills’ (Dinham, 2006, p. 847). Such ideas build on the concept of andragogy (Knowles, 1972, 1998) whereby mutual processes of learning and growth are encouraged.

The knowledge review conducted by Trevithick et al. (2004) identified articles where the theoretical foundations for teaching skills in social work were made explicit. The communication skills module at the University of York in the UK, based on Agazarian's theory, is located within a systems framework (Koprowska, 2003). Relational teaching based on relational/cultural theory should underpin teaching in social work education, whereby mutual engagement, mutual empathy, and mutual empowerment foster growth in relationships between tutors and students (Edwards & Richards, 2002). These examples are the exception to the rule; few articles theorise the teaching and learning process (Eraut, 1994). Generally speaking, ‘communication skills have been taught, but not reflected upon; experienced, but not theorised’ (Moss et al., 2007, p. 711).

A wide variety of approaches for teaching communication skills to social work students exist in practice. Given there is more expertise in the teaching and learning of communication skills than the literature denotes, academics should continue theorising and researching this aspect of the curriculum (Dinham, 2006). Although rigorous high‐quality evaluation of outcomes in social work education is still in the early stages of development (Carpenter, 2011), teaching communication skills to social work students is an aspect of the curriculum which has attracted considerable attention, therefore a review of the findings can help to clarify what we know about this important topic.

### 2.4 Why it is important to do this review

A variety of communication skills courses have been proposed and are in use in social work education. It is nearly twenty years since a number of practice and knowledge reviews highlighted the lack of evaluation into communication skills courses, an issue which warranted further research (Dinham, 2006; Trevithick et al., 2004). To support this endeavour, methodological guidance for evaluating outcomes in social work education (Carpenter, 2005, 2011) has been produced. Consequently, a number of empirical studies (Koprowska, 2010; Lefevre, 2010; Tompsett, Henderson, Gaskell Mew, et al., 2017) have sought to evaluate the teaching of communication skills among social work students, or investigate the impact of particular components of the intervention. Existing literature suggests that teaching social work students communication skills increases their self‐efficacy in terms of communicative abilities (Koprowska, 2010; Lefevre, 2010; Tompsett et al., 2017). Good communication is fundamental to effective social work practice.

No comprehensive systematic review or meta‐analysis of this aspect of social work education has been undertaken; questions concerning whether the teaching of communication skills to social work students is effective and produces positive outcomes remain unanswered. It is time therefore to identify, summarise and synthesise the empirical research into a systematic review. Doing so will form a reliable, scientifically rigorous, and accessible account that can be used by educators and policy‐makers to guide decisions about which approaches are effective in teaching communication skills to social work students. In this time of political uncertainty and financial constraint, ‘it is important to accumulate evidence of the outcomes of social work education so that policy‐makers and the public can be confident that it is producing high‐quality social workers’ (Carpenter, 2016, p. 192), who are suitably equipped to deal with the demands of social work practice. We conducted this systematic review to determine whether CST for social work students works and which types of CST, if any, were the most effective and lead to the most positive outcomes. To improve uptake and relevance, the systematic review was developed in consultation with stakeholders (including academics, students, practitioners, and people with lived experience) and advice was sought from leading social work organisations. The review also sheds light on areas where more research is required.

## 3 OBJECTIVES

This systematic review aimed to critically evaluate all studies which have investigated the effectiveness of CST programmes for social work students. The PICO (Population, Intervention, Comparator, Outcomes) framework and stakeholder collaboration informed the development of the research question. Student social workers constituted the population, CST was the intervention under investigation, the absence of CST or a course unrelated to communication were the comparators, and attitudes, knowledge, confidence and behavioural changes were the outcomes of interest. Stakeholders had agreed that neither the comparator nor the outcomes should be specified within the research question itself, on the grounds that researchers and academics were unlikely to have specified these elements in the primary studies. The review built on an existing knowledge review (conducted by Trevithick et al., 2004) but was not restricted by the year of publication or language. The research question which the review posed is: ‘What is the effectiveness of CST for improving the communicative abilities of social work students?’ It was intended that the review would provide a robust evaluation of CST for social work students and explain variations in practice. To test the effectiveness of interventions, hierarchies of evidence point to systematic reviews of (preferably randomised) controlled trials. Therefore, we sought to conduct a rigorous and systematic review of such studies about CST, supporting educators and policy‐makers to make evidence‐based decisions in social work education, practice and policy.

The protocol for this review was published in the Campbell Collaboration Library (Reith‐Hall & Montgomery, 2019).

## 4 METHODS

### 4.1 Criteria for considering studies for this review

#### 4.1.1 Types of studies

The studies were required to include an appropriate comparator to be eligible for inclusion in the review, irrespective of whether outcome data were reported in a useable way. Permitted study designs included: randomised trials, non‐randomised trials, controlled before‐after studies, repeated measures studies and interrupted time series studies. To be included, interrupted time series studies needed a clearly defined point in time when the intervention occurred and at least three data points before and three after the intervention. The justification for this wider range of study types was to identify any potential risk of harm which we hoped to assess through wider evidence. Potential risk of harm included any negative effects of CST on students’ communicative abilities, for example, service users and carers might have indicated that students’ poor communication left them feeling more confused, agitated, misunderstood or distressed (i.e., worse) than they did before the interaction.

To ensure quality of evaluation, all studies were critically appraised and an analysis of the results by study design was considered. The comparison group were composed of those who received no educational intervention or those receiving educational interventions other than CST. Trials comparing the effects of two different educational interventions to improve communication skills were also included in this review. In accordance with Campbell policies and guidelines (The Campbell Collaboration, 2014), studies without comparison groups or appropriate counterfactual conditions were excluded.

#### 4.1.2 Types of participants

All social work students who were taught communication skills on a generic qualifying social work course in a university setting were included hence undergraduate and postgraduate students were among the types of participants. Students on post‐qualifying courses were excluded.

#### 4.1.3 Types of interventions

Only studies in which the intervention group received CST and in which the control group received nothing or received an alternative training to the intervention group were included. For the intervention, any underpinning theoretical model and any mode of teaching (taught input, videotape recording, role‐play with peers, simulated interviews with service users and carers or actors) were considered acceptable. Interventions that took place either entirely or predominantly in a university setting were included.

#### 4.1.4 Types of outcome measures

Outcomes included changes in (1) knowledge, (2) attitudes, (3) confidence/self‐efficacy and (4) behaviours measured using objective and subjective scales. It was anticipated that these measures might be study‐specific rating scales, developed for use in evaluating communication skills. Stakeholder involvement indicated that behavioural change was an important outcome for all stakeholders. In addition, students and educators deemed confidence/self‐efficacy to be a relevant outcome. In keeping with the literature on outcomes in social work education (Carpenter, 2005, 2011), student satisfaction alone was not considered as an outcome measure in this review.

### 4.2 Search methods for identification of studies

We conducted a search for published and unpublished studies using a comprehensive search strategy informed by the guide to information retrieval for Campbell systematic reviews (Kugley et al., 2017). We also sought advice from information specialists. Our search strategy included searching multiple electronic databases, research registers, grey literature sources, and reference lists of prior reviews and relevant studies. Study selection was not restricted by geography, language, publication date or publication status. The original search took place in September 2019 and an updated search took place in June 2021.

#### 4.2.1 Electronic searches

To identify eligible studies the following data sources were searched using the search strings set out in Supporting Information: Appendix A:

(a) Education Abstracts (EBSCO)
(b) ERIC (EBSCO)
(c) MEDLINE (OVID)
(d) PsycINFO (OVID)
(e) Web of Science/Knowledge Database Social Science Citation Index
(f) Social Services Abstracts (Proquest)
(g) ASSIA—Applied Social Sciences Index and Abstracts (Proquest)
(h) ClinicalTrials.gov
  Relevant reviews were searched for in the following databases:
(i) Database of Abstracts of Reviews of Effectiveness
(j) The Campbell Library
(k) Cochrane Collaboration Library
(l) Evidence for Policy Practice Information and Coordinating Centre (EPPI‐Centre)
  We also searched grey literature, using the following databases and websites:
(m) Google Scholar—using a series of searches, the first 2 pages of results for each search were screened
(n) ProQuest Dissertations and Theses

#### 4.2.2 Searching other resources

We searched for conference proceedings and abstracts through Web of Science and ERIC, followed by a Scopus search which did not unearth any new sources of information. We also looked at generic websites including Google and Bing as well as government websites and professional websites such as gov.uk and the department for education, the Higher Education Academy, British, Australian and American Councils/Associations of Social Work and Social Work Education, Community Care and the Social Care Institute for Excellence website, which includes Social Care online. Several searches containing the key words used in the database searches were replicated for these additional sources.

We also searched the reference lists of the included studies and relevant reviews to identify additional studies. Prominent authors were contacted for further information about their studies and asked if they were aware of any other published or ongoing studies meeting our inclusion criteria. In addition, a final step towards the end of analysis, a manual search of the most recent issue(s) of five key journals that provided relevant studies were identified and checked. These were the Journal of Social Work Education, Social Work Education, the British Journal of Social Work, Children and Youth Services Review and Research on Social Work Practice.

### 4.3 Data collection and analysis

We collected and analysed data according to our protocol (Reith‐Hall & Montgomery, 2019).

One reviewer (ERH) conducted the database searches, removing duplicates and irrelevant records. Having anticipated that the searches would result in very few records to screen, each record was screened by title and abstract by both reviewers (ERH and PM), to ensure robustness. Any studies deemed to be potentially eligible were retrieved in full text and screened by both reviewers. There were no disagreements hence discussions with an arbitrator was not required and consensus was reached in all cases.

The search strategy was developed using the terms featuring in existing knowledge and practice reviews and in consultation with social work researchers and academics, to ensure that the broad range of terminology was included. Search strings included terms relating to the intervention and population but not study design. A sample search strategy for Medline can be found in Supporting Information: Appendix A. Search strings and search limits were modified for each database. Proximity searching was not required.

#### 4.3.1 Selection of studies

Included studies were any form of design where appropriate counterfactual conditions were satisfied, in accordance with the Cochrane Effective Practice and Organisation of Care guidelines for the inclusion of non‐randomised studies (Cochrane EPOC, 2017).

To ensure that the effects of an individual intervention were only counted once, we anticipated applying the following conventions: (1) Where there were multiple measures reported for the same outcome, an average effect size for each outcome would be calculated within each study. (2) Where the same outcome construct was measured across multiple time domains, the main analysis would focus on synthesising the evidence relating to effect sizes at immediate post‐test. Any subsequent measures of outcomes beyond immediate post‐test would be analysed and reported separately.

#### 4.3.2 Data extraction and management

Once eligible studies were found, an initial analysis of intervention descriptions was undertaken for each. The Campbell data collection template form was used to identify the core components of programmes and to develop an overarching typology and coding frame.

*Details of study coding categories*

Components included:

- Duration and intensity of the programme.
- Whether programme delivery included people with lived experience (e.g., service users and carers)
- Whether programmes used audio and video recording
- Whether communication skills were practised with peers, service users or actors
- Whether programmes included observation of social workers in practice
- The theoretical frameworks underpinning the intervention

Alongside extracting data on programme components, descriptive information for each of the studies was extracted and coded to allow for potential sensitivity and subgroup analysis. This included information regarding:

- Study characteristics in relation to design, sample sizes, measures and attrition rates.
- Whether the study was conducted by a research team associated with the programme or an independent team.
- Stage of programme development, for example whether it was a new programme being piloted or an established programme being replicated.
- Participants’ characteristics in relation to age, sex, ethnicity, geo‐political region and socio‐economic background.

We considered subgrouping the different types of intervention and population, based on factors such as length of course and teaching methods, age and sex, however the small number of included studies did not warrant subgroup analysis.

Coding was carried out by the review team independently; discrepancies were discussed, and a consensus reached.

Quantitative data was extracted to allow for calculation of effect sizes (using mean change scores and post‐test means and standard deviations). Data was extracted for the intervention and control groups on the relevant outcomes measured to assess the intervention effects.

#### 4.3.3 Assessment of risk of bias in included studies

Assessment of methodological quality and potential for bias was conducted using the Cochrane Risk of Bias tool for randomised studies (Higgins et al., 2019) and the ROBINS‐I tool for non‐randomised studies (Sterne, Higgins, et al., 2016; Sterne, Hernán, et al., 2016).

#### 4.3.4 Measures of treatment effect

Continuous outcomes were reported by the included studies, so we used the standardised mean difference (SMD) as our effect size metric where means and standardised deviations were provided by study authors. Where means and standard deviations were not available, we calculated SMDs from *t*‐values and calculated standard deviations from standard errors where these were provided using recommended methods (Higgins et al., 2022). Hedges’ *g* was used for estimating SMDs to correct for the bias associated with small sample sizes. In studies with more than two groups, we calculated effect sizes using the experimental and control groups that were most relevant to answering our research question or used data from groups with the largest numbers in them.

##### Treatment of qualitative research

This systematic review was limited to synthesising the available evidence on the effectiveness of CST to social work students. It was beyond the remit of this present review to synthesise the associated evidence related to process evaluations of such programmes hence we did not include qualitative research.

#### 4.3.5 Unit of analysis issues

The unit of analysis for this review was social work students. No unit of analysis issues were identified for the included studies.

#### 4.3.6 Dealing with missing data

Study authors were contacted and accompanying or linked papers were sought in an effort to retrieve missing data.

#### 4.3.7 Assessment of heterogeneity

Widespread clinical heterogeneity within the included studies rendered other anticipated measures of treatment effect non‐viable. For example, the included populations consisted of undergraduate, postgraduate, mixed and unreported students, whilst the interventions differed according to duration, uptake, mode and key features. Widespread methodological diversity was present in terms of designs, methodologies, and outcome measures across studies.

#### 4.3.8 Assessment of reporting biases

Reporting was generally poor among the included studies as evidenced by limited use of reporting instruments such as CONSORT and no references to pre‐published protocols were made by study authors. A more detailed discussion of this issue can be found in the Risk of Bias section. Use of a funnel plot, which helps to identify potential reporting bias in the included studies was not feasible, given the small number of studies included in this review.

The use of a highly sensitive and inclusive systematic search of bibliographic databases, grey literature sources, reference list searching, correspondence with study authors and hand searching sought to counteract potential bias in our reporting of this review.

#### 4.3.9 Data synthesis

As a result of this heterogeneity, meta‐analysis was not feasible, nor was it possible to implement methods outlined in the protocol, such as sensitivity and subgroup analysis. I_2_ and Tau_2_ were not measured or reported in this review. Similarly, we were unable to use the new GRADE Guidance for Complex Interventions (unpublished) to summarise the overall quality of evidence relating to the primary outcomes.

#### 4.3.10 Subgroup analysis and investigation of heterogeneity

N/a in view of there being no meta‐analysis.

#### 4.3.11 Sensitivity analysis

N/a in view of there being no meta‐analysis.

##### Summary of findings and assessment of the certainty of the evidence

N/a

## 5 RESULTS

### 5.1 Description of studies

There are 15 studies included in this review. An overview of the key characteristics of the included studies, which are described in terms of study design, participants, interventions, comparators, outcomes, outcome measures, geographical location, publication status and implementation factors are provided in Table 1.

**Table 1:** Included studies table.

First author, date	Study design	*N*	Population	Intervention	Comparator	Outcomes	Measures	Location	Publication status	Implementation factors (e.g., amount, duration)
Barber, 1988 Experiment 1	Case control	32	Undergraduate social work students Male, *N* = 8 (25%) Age range: 19‐46 Mean age: 25.7	Microskills training *N* = 16 final year students	No training *N* = 16 first year students	Counsellor: Trustworthiness Attractiveness Expertness Non‐verbal communication	Counselor Rating Form (Barak & LaCrosse, 1975). Non‐verbal rating skills	La Trobe, Victoria, Australia	Published journal article	Amount: ‘extensive’ Duration: 4‐year programme
Barber, 1988 Experiment 2	Case control	50	Undergraduate social work students Population characteristics not stated	Microskills training *N* = 25 final year students	No training *N* = 25 first year students	Trustworthiness Attractiveness Expertness	Counselor Rating Form (Barak & LaCrosse, 1975). Non‐verbal rating skills	La Trobe, Victoria, Australia		Amount: ‘extensive’ Duration: 4‐year programme
Collins, 1984	Case control	67	Masters level social work students	Skills lab training course (*N* = 54) Age range: 21–43 Mean age: 26.78 Male, *N* = 9 (17%)	Lecture‐based training course (*N* = 13) Male, *N* = 6 (46%)	Interviewing skills; empathy, warmth, genuineness	Skills acquisition measure (SAM) Carkhuff's communication index Analogue interview Client interview	University of Toronto, Canada	Dissertation thesis	Amount: not stated Duration: 2 months
Greeno, 2017	RCT	54	Undergraduate and master's level social work students Male, *N* = 8 (15%) 51% (*N* = 28) Caucasian 45% (*N* = 24) Black 4% (*N* = 2) Hispanic Age range: 20–55 Mean age: 29.7	Live supervision with standardised clients	TAU—online self‐study	Perceived empathy Empathic behaviour	Motivational Interviewing Treatment Integrity; Toronto Empathy Questionnaire	University of Maryland, Baltimore, MD, USA	Published journal article	Amount: 3 days (6 h of didactic teaching, followed by 2 days of Live Supervision or 2 days of online learning) Duration: unstated (study took place over 7 months, which included 5‐month follow‐up
Pecukonis, 2016						MI skills, adherent behaviours and proficiency level Self‐efficacy	Motivational Interviewing Treatment Integrity coding system; general self‐efficacy scale		Published journal article	
Hettinga, 1978	RCT	38	Masters social work students (*N* = 34) Undergraduate students (*N* = 1) Male, *N* = 7 (18%) Age range: 22‐45 Mean age: 31.3	Communication skills training using videotaped interview playback with instructional feedback *N* = 23 (3 did not complete measures)	Communication skills training (face to face) with group feedback *N* = 15	Self‐esteem Self‐perceived interviewing competence	Rosenberg Self‐Esteem Scale Self‐Perceived Interviewing Competence Questionnaire	University of Minnesota, USA	Dissertation thesis	Amount: 3 h per week Duration: 1 quarter of an academic year
Keefe, 1979	Case control	56	Second year master's social work students Population characteristics not stated.	(1) a course of instruction with both experiential and didactic content *N* = 19 (2) a structured meditation series *N* = 20.	TAU *N* = 17	Empathic skill	Kagan's Affective Sensitivity Scale	The University of Utah	Published journal article	Instruction Amount: 2.5 h per week Duration: 1 quarter of an academic year Zen meditation Amount: 30 min per day Duration: 3 weeks
Larsen, 1978	RCT	94	First year master's social work students Population characteristics not stated.	Communication laboratories consisting of didactic and experiential learning *N* = 59	Traditional didactic instruction *N* = 35	Facilitative conditions (empathy, non‐possessive warmth, genuineness)	The Index of Therapeutic Communication	The University of Utah	Published journal article	Amount: 10 h Duration: Not stated
Laughlin, 1978	RCT	78	Undergraduate social work students Male: *N* = 11 (14.1%) Age range: 20‐59 Mean age: 23.4 Median age of 21.	(1) Experimental Group I: self‐instruction manual plus audio practice tapes with supervisor evaluation, feedback, and reinforcement (2) Experimental Group II: self‐instruction manual plus audio practice tapes with self‐evaluation and self‐reinforcement	(3) Control Group I: introductory section of self‐instruction manual, expectation set, and instructions to practice (4) Control Group II: no instructional materials.		Revised version of the Carkhuff Communication Index (Carkhuff, 1969a). Carkhuff's empathic understanding scale (Carkhuff, 1969a)	University of California at Berkeley.	Dissertation thesis	Amount: Not stated but experimental groups participated in 3 lab sessions. Duration: 2 weeks
Ouellette, 2006	Case control	30	Undergraduate social work students Male, *N* = 2 (6.7%) Age range: 20–40+ Mean age: Not specified Age 20–29, 53.3% Age 30 and 39, 30% Age 40+, 16.6% 60% (*N* = *N* = 18) Caucasian 33.% (*N* = 10) African American 3.3% (*N* = 1) Hispanic 3.3% (*N* = 1) ‘Other’.	Online *N* = 16	Classroom *N* = 14	Basic interviewing skills	Basic practice interviewing skills scale	Indiana University	Published journal article	Amount: 1 × 3‐h session per week Duration: 15 weeks
Rawlings, 2008	Case control	32	Undergraduate social work students Male, *N* = 2 (6.3%) Age range: Not specified Mean age: 20.81 78% (*N* = 25) Caucasian 10% (*N* = 3) Hispanic 6% (*N* = 2) Biracial 3% (*N* = 1) African American 3% (*N* = 1) ‘Other’.	Exiting social work students (*N* = 16)	Starting SW students (*N* = 16)	Self‐efficacy Skill performance	Social Work Direct Practice Self‐Efficacy Scale basic practice skill performance (Chang & Scott, 1999) Three item direct practice skill sub‐scale reflecting core conditions for each student	Case Western Reserve University	Dissertation	Amount: not stated Duration: BSW degree
Schinke, 1978	RCT	23	Graduate social work students Males *N* = 7 (30.4%) Age range: Not stated Mean age: 29.87	Interviewing skills *N* = 12	Delayed start control group *N* = 11	Attitudes towards their own role‐played interviewing behaviour	Videotaped interview ratings Counselor effectiveness scale developed by Ivey (1971)	University of Washington	Published journal article	Amount: 4 h Duration one‐off session
Toseland, 1982	Case control	68	Undergraduate social work students (*N* = 55) Undergraduate social welfare students (*N* = 13) Population characteristics not stated.	Interpersonal skills training (*N* = 55)	13 social welfare students—no skills training (*N* = 13)	Ten interpersonal helping skills	The Carkhuff Indices of Communication and Discrimination and the Counseling Skills Evaluation Parts 1 and 2	Not stated	Published journal article	Amount: 15 × 2 sessions in the lab plus lectures (Total of 45 h) Duration: one semester
VanCleave, 2007	Case control	45	Masters level social work students Age range: early twenties to mid fifties Mean age: Not stated. Age 20‐25, 35% (*N* = 16) Age 26‐30, 27% (*N* = 12) Age 31‐35, 11% (*N* = 5) Age 35+, 27% (*N* = 12) Male *N* = 3 (6.6%) 95% (*N* = 43) Caucasian, 2% (*N* = 1) African American 2% (*N* = 1) Japanese	Additional empathy training (*N* = 22)	TAU (*N* = 23)	Empathic response Perspective taking and empathic concern	Carkhuff's Index for Communication scripts (CIC) A 14‐question, self‐survey for Empathic Concern (EC) and the Perspective Taking (PT) subscales of the Davis (1980) Interpersonal Reactivity Index (IRI).	University of Southern Indiana	Dissertation thesis	Amount: 10 h Duration: within a 3‐month cycle
Vinton, 1994	Case control	62	Undergraduate social work students Age range: 19–54 Mean age: 25.9 Male *N* = 7 (11.3%)	Videotape other Videotape other + self	Delayed start control group	Perceived empathy Empathy	Questionnaire Measure of Emotional Empathy (QMEE) Carkhuff's level of empathy scale	Florida State University	Published journal article	Amount: not stated but includes a 100‐min standardised lecture. Duration: not stated
Wells, 1976	RCT	14	Social work students (type not specified) Population characteristics not stated.	Role‐play	Own problems	Empathy	Carkhuff's empathic understanding scale	University of Pittsburgh	Published journal article	Amount: 1 day of didactic training, 6 × 2‐h sessions of experiential learning Duration: not stated

### 5.2 Results of the search

The main bibliographic database and registers search, completed in September 2019, returned 1998 records with an additional 12 added after the search was updated in June 2021. After 882 duplicate records were removed, 1128 were subjected to initial screening by title, and abstract if necessary, following which a further 1021 records were removed because they were not relevant to the topic. Of the 107 remaining records, 2 could not be retrieved despite endeavours to locate them through different libraries and searches, therefore 105 records were fully screened for eligibility, 9 of which met the inclusion criteria.

Another 650 studies were identified through recent editions of five key journals identified through the database search. A further 19 studies were identified through other methods including citation searching within the included studies. Of the 669 studies subjected to initial screening, 627 were removed because they were not relevant to the topic. One record could not be retrieved resulting in 41 records being fully screened for eligibility, of which 34 records were excluded, and 7 records (reporting 6 studies) were included.

Of the fifteen studies which met the inclusion criteria for this systematic review, two experiments are reported in a single paper (Barber, 1988), one study is reported in two papers (Greeno et al., 2017; Pecukonis et al., 2016)‐with both authors contributing to the write‐up of each, and another study (Larsen & Hepworth, 1978) is also written up as the first author's PhD thesis (Larsen, 1975).

**Figure 1:**
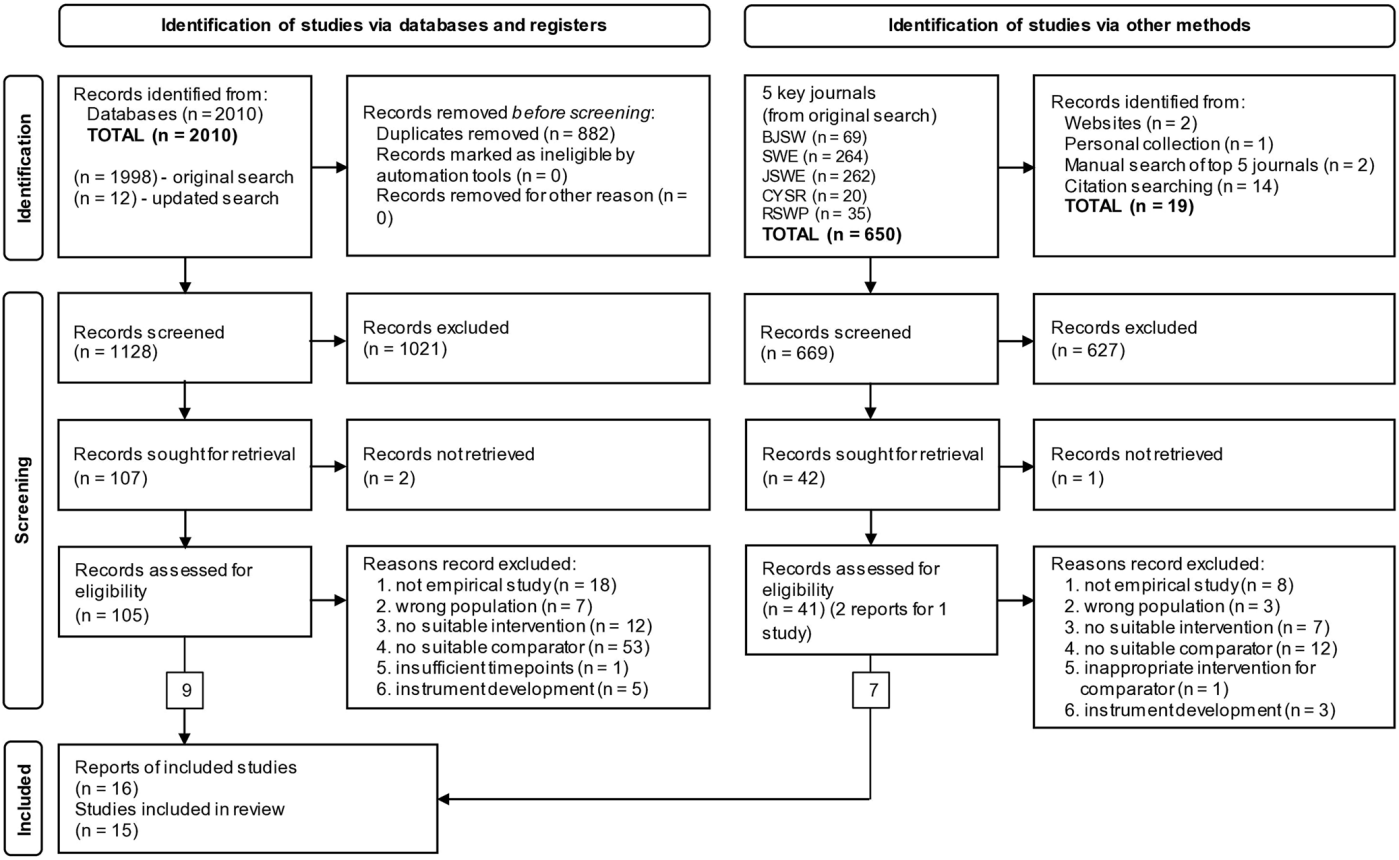
PRISMA diagram.

The search results are shown in the PRISMA diagram (adapted from Page et al., 2021) in Figure 1.

#### 5.2.1 Included studies

##### Study design characteristics

Despite the varied terminology used by the study authors to describe their research designs, eight reports, addressing nine studies (Barber, 1988; Collins, 1984; Keefe, 1979; Ouellette et al., 2006; Rawlings, 2008; Toseland & Spielberg, 1982; VanCleave, 2007; Vinton & Harrington, 1994) employed a case‐controlled design, some of which conform to the parameters of a pre‐experimental static group comparison design (Campbell & Stanley, 1963). This means that participants were divided between two groups but in a non‐randomised way. Given students were not randomised to the different groups, these studies suffer from weak internal validity, with confounders such as maturation, the Hawthorne effect, testing effects and pre‐existing differences between the intervention and control groups. Such issues are common in educational research.

Six of the studies reported in seven papers were randomised controlled trials (RCTs), five of which were conducted in the mid to late 1970s. The increase of research activity surrounding this topic during this decade likely results from the development of teaching models such as Ivey and Authier's micro‐counselling model (Ivey & Authier, 1971; Ivey et al., 1968) and the Truax and Carkhuff Human Relations training model (Carkhuff, 1969c; Truax & Carkhuff, 1967), alongside the development of research measures, including the Carkhuff scales (Carkhuff, 1969a, 1969b), which are the most cited research instrument in this review.

Wells (1976) is the earliest of the included studies to use an RCT design, comparing role‐play and students’ ‘own problem’ procedures, but the sample size contained just 14 students. Hettinga (1978) had a somewhat larger sample of 38 students, in which immediate feedback from an instructor was compared with group feedback provided later. Although quasi‐randomisation took place, it is unlikely the allocation method affected the results. In the same year, Larsen and Hepworth (1978) investigated the role of experiential learning; controls received traditional didactic instruction. Schinke et al. (1978) randomly allocated a group of 23 students to either an intervention group or a waiting‐list control. Laughlin (1978) used a more complex design consisting of two experimental groups and two control groups. Despite using pre‐tests, a strategy which can help overcome methodological challenges associated with small sample sizes (social work cohorts are typically small), the study was hopelessly underpowered. The most recent of the included studies, reported in the two papers by Pecukonis et al. (2016) and Greeno et al. (2017), offers the most robust research design of the included studies. Not only did they exceed the minimum sample size calculated in an a priori power analysis, but the overall risk of bias was lower than other studies included in this review.

In terms of comparators, in four of the studies the control group received no intervention (Barber, 1988; Rawlings, 2008; Toseland & Spielberg, 1982); three studies reported controls receiving treatment as usual (TAU) (Greeno et al., 2017; Keefe, 1979; Pecukonis et al., 2016; VanCleave, 2007), however the TAU in Greeno and Pecukonis’ study was an online intervention, as opposed to an absence of an intervention; and a further five studies compared two different interventions. These included an experiential approach with traditional didactic learning (Larsen & Hepworth, 1978); lab‐based versus lecture‐based training (Collins, 1984); online versus classroom‐based teaching (Ouellette et al., 2006); videotaped interview playback with instructional feedback versus peer group feedback (Hettinga, 1978); and role‐play versus students’ ‘own problems’ procedures (Wells, 1976). In a rather complex design, Laughlin 1978 study included two treatment arms, and two control groups, one of which received no treatment. In two subsequent studies (Schinke et al., 1978; Vinton & Harrington, 1994), the controls had a delayed start (operating as a waiting list procedure).

Significant issues with measurement are evident within the included studies and are acknowledged by several of the researchers (Collins, 1984; Greeno et al., 2017; Laughlin, 1978; Vinton & Harrington, 1994). Methodological challenges will be considered in Section 6.

##### Publication status

Five of the studies were dissertation theses (Collins, 1984; Hettinga, 1978; Laughlin, 1978; Rawlings, 2008; VanCleave, 2007), with the remainder being reported in peer reviewed journals (Barber, 1988; Greeno et al., 2017; Keefe, 1979; Larsen & Hepworth, 1978; Ouellette et al., 2006; Pecukonis et al., 2016; Schinke et al., 1978; Toseland & Spielberg, 1982; Vinton & Harrington, 1994; Wells, 1976).

##### Population characteristics

A total of 743 research participants were contained within the 15 included studies. Of the included studies, seven studies (reported in Barber, 1988; Laughlin, 1978; Ouellette et al., 2006; Rawlings, 2008; Toseland & Spielberg, 1982; Vinton & Harrington, 1994) contained undergraduate students (*N* = 352) and five studies (Collins, 1984; Keefe, 1979; Larsen & Hepworth, 1978; Schinke et al., 1978; VanCleave, 2007) comprised Master's social work students as their participants (*N* = 285). One study (Wells, 1976) failed to specify student type (*N* = 14) whilst two studies (Greeno et al., 2017; Hettinga, 1978; Pecukonis et al., 2016) used a combination of undergraduate and Master's students (*N* = 92).

###### Sex

Ten of the included studies report on the number and percentage of men and women in the student samples. In Collins' (1984) study, of the 54 students in the lab group, 17% (*N* = 9) were men, however of the 13 students from the lecture group sample, 46% (*N* = 6) were men; the number of men in the lecture group was unusually high. Collins (1984, p. 74) acknowledges this is not explained by the admissions procedures at either of the universities involved in the study. However, it must be remembered that the 13 students from the lecture group, who volunteered to be part of the study, are not necessarily representative of the cohort demographic.

A more consistent picture is evident amongst the other studies, in which men make up less than a third of the social work students in the samples, reflecting a demographic pattern found among qualified social workers. The number and percentage of men in the student samples (arranged in ascending order by percentage) were as follows: 6% (*N* = 2) for Rawlings (2008); almost 7% for both Ouellette et al. (2006) and VanCleave (2007) (*N* = 2 and *N* = 3, respectively); 11% (*N* = 7) for Vinton and Harrington (1994); 14% (*N* = 11) for Laughlin (1978); 15% (*N* = 8) in the study reported by Pecukonis et al. (2016) and Greeno et al. (2017); 18% (*N* = 7) for Hettinga (1978); 25% (*N* = 8) in Barber (1988) ‐ experiment 1 and just over 30% (*N* = 7) in the study conducted by Schinke et al. (1978). The sex of students was not reported in five of the studies (Barber, 1988‐experiment 2; Keefe, 1979; Larsen & Hepworth, 1978; Toseland & Spielberg, 1982; Wells, 1976).

###### Age

Due to differences in reporting practices, the age characteristics of the students in the included studies are harder to compare. In the same five studies identified above (Barber, 1988‐experiment 2; Keefe, 1979; Larsen & Hepworth, 1978; Toseland & Spielberg, 1982; Wells, 1976), age characteristics were not reported.

The age range was not specified in Rawlings' (2008) study, although students had the lowest mean age of 20.8 (18.8 for entering students and 22.9 for exiting students). The mean age of students in Laughlin's study was 23.4, with the broadest age ranges of 20‐59. In Barber's (1988) paper, for experiment 1 the ages ranged from 19 to 46 years, with a mean age of 25.7 years. With a slightly broader age range of 19–54, students in Vinton and Harrington's (1994) study had a mean age of 25.9. In Collins' (1984) study, the ages of the lab trained students ranged from 21 to 43 years, with a mean age of 26.7; the lecture trained students are described as being ‘slightly older’ (p. 74). The age range for the students in the study reported by Pecukonis et al. (2016) and Greeno et al. (2017) was 20–55, with a mean age of 29.7. An age range was not specified in Schinke et al's. (1978) study, although the mean age was 29.87. Of the studies where data about mean age were available, students in the study undertaken by Hettinga, 1978 had the oldest mean age of 31.3, with an age range of 22–45. In Ouellette et al's. (2006) study, an age range of 20–40+ is reported. A mean age is not provided, however 53.3% of students were between the ages of 20 and 29, 30% were between the ages of 30 and 39, and 16.6% were older than 40. In keeping with the age ranges of the other studies, the age range in VanCleave's (2007) study was described as early twenties to mid‐fifties. No mean age was provided, however 35% (*N* = 16) of students were between the ages of 20 and 25, almost 27% (*N* = 12) were between 26 and 30, 11% (*N* = 5) were between the ages of 31 to 35 and almost 27% (*N* = 12) were over 35 years.

###### Ethnicity

Only the four studies conducted since 2000 reported information on ethnicity, in the following ways: In the study conducted by Ouellette et al. (2006), 60% (*N* = 18) of students were Caucasian, 33.3% (*N* = 10) were African American, 3.3% (*N* = 1) were Hispanic, and 3.3% (*N* = 1) identified as ‘Other’. Rawlings (2008) identified that 78% of students (*N* = 25) were Caucasian, almost 10% (*N* = 3) were Hispanic, just over 6% (*N* = 2) were Biracial, 3% (*N* = 1) were African American and 3% (*N* = 1) were defined as ‘Other’. In the study reported by Pecukonis et al. (2016) and Greeno et al. (2017), just over 51% (*N* = 28) of students were Caucasian, 45% (*N* = 24) were Black and almost 4% (*N* = 2) were Hispanic. In VanCleave's (2007) study, over 95% (*N* = 43) of students were Caucasian, one student was African American and one was Japanese—each accounting for just over 2%. The earlier studies did not report on the ethnicities of their participants, reflecting changes to trends in the collection of demographic data.

Data is absent for other demographic characteristics within the included studies.

##### Location characteristics

There is little variation within the geo‐political contexts in which the included studies were conducted. This is important because it reflects some priorities such as the primacy placed on experimental design, at the expense of others, including stakeholder involvement. One study, Collins (1984) (*N* = 67) was undertaken in Toronto, Canada, whilst Barber (1988) reports on two experiments conducted in Victoria, Australia (*N* = 82). One study, Toseland and Spielberg (1982) did not provide a location (*N* = 68). The remaining 11 studies were carried out in different US states, where the focus on evidence‐based teaching and learning in social work education is firmly established. Involvement and participation from people with lived experience was noticeably absent—the second of the Barber (1988) experiments and the client interviews in Collins' (1984) study being the exceptions. None of the included studies were conducted in the UK, where a strong tradition of service user and carer involvement in social work education prevails, which arguably explains, but does not justify, the omission of contributions from people with lived experience within the body of research identified in this review.

##### Intervention characteristics

###### Theoretical orientation

Experiential learning is referred to in the majority of the studies (Collins, 1984; Greeno et al., 2017; Keefe, 1979; Larsen & Hepworth, 1978; Laughlin, 1978; Pecukonis et al., 2016; Rawlings, 2008; Schinke et al., 1978; Toseland & Spielberg, 1982) as the underpinning theoretical orientation of the intervention under investigation. However, the term is not applied consistently. With its wide range of different meanings, ideologies, methods and practices, experiential learning is conceptually complex and difficult to define (Moon, 1999). Conceptualisations arising from two different traditions are evident within the included studies: first, the work of Carkhuff and Truax (1965) and Ivey and Authier (1971), which derive from psychotherapy, and second, the work of Kolb (1984) and Schön (1987) which is grounded in a constructivist view of education and has been particularly instrumental within professional courses.

Although deriving from psychotherapy, the microskills counselling approach developed by Ivey et al. (1968) and Ivey and Authier (1971) has informed the teaching of interviewing skills in social work education. Content comprises well‐defined counselling skills including attending behaviour, minimal activity responses, and verbal following behaviour. Six of the included studies made reference to the work of Ivey and colleagues, however five of them (Collins, 1984; Hettinga, 1978; Laughlin, 1978; Rawlings, 2008; VanCleave, 2007) did so simply within a discussion of the wider literature. It is only in Schinke et al.'s (1978) study where Ivey's work has a direct impact on the empirical evaluation itself; an adapted version of the Counsellor Effectiveness Scale developed by Ivey and Authier (1971) was used as one of the study's measuring instruments.

Referred to as the Human Relations training model, the work of Carkhuff and Truax (1965) and Carkhuff (1969c) has been more influential than Ivey's approach. A brief exploration of empathy as a theoretical construct helps to explain why Carkhuff and Truax's work has influenced social work education and practice. Whilst linguistic relevance can be seen in the Greek word ‘empatheia’, which means appreciation of another's pain, the philosophical underpinnings of the term empathy actually derives from the German word Einfühlung. Theodor Lipps expanded the conceptualisation of empathy to include the notion of minded creatures, of which inner resonance and imitation are a part. Lipps’ ideas influenced how empathy came to be understood in psychotherapy and is evident in the work of Sigmund Freud and Carl Rogers. Empathy was identified by Rogers (1957) as one of ‘the necessary and sufficient conditions’ for therapeutic personality change; his ideas about person‐centred practice remain central to social work education and practice today. Charles Truax, a protégé of Rogers, worked closely with Robert Carkhuff, to explore how conceptual orientations such as empathy could be observed, repeated, measured and taught. Carkhuff and Truax (1965) developed and evaluated an integrated didactic and experiential approach in a counselling and psychotherapy context, which ‘focuses upon therapist development and growth’ (p. 333). Their work, and the ideas that influenced them, are evident throughout the earlier studies of this review where empathy was the focus. Barber (1988) cited Carkhuff's work on empathy in his discussion of the literature, whilst Keefe (1979) referred to it for teaching purposes only. Seven studies (Collins, 1984; Larsen & Hepworth, 1978; Laughlin, 1978; Toseland & Spielberg, 1982; VanCleave, 2007; Vinton & Harrington, 1994; Wells, 1976) used the Carkhuff scales (Carkhuff, 1969a; 1969b) as an outcome measure in their empirical research. As identified by Elliott et al. (2018), the Carkhuff scales were some of the earliest observer measures, which may well explain the popularity of this instrument. The focus the researchers of the included studies placed on empathy is striking and will be considered further in subsequent sections.

Also apparent in the literature is the experiential learning approach deriving from the experiential learning cycle developed by Kolb (1984) and the concept of reflective practice articulated by Schön (1987). Rawlings (2008), who provides the most comprehensive overview of experiential learning in the included studies, draws on the work of both. Huerta‐Wong and Schoech (2010) suggest that experiential learning has been a teaching technique used extensively to teach social workers skills in the United Kingdom and the United States since the 1990s. They explain that ‘experiential learning proposes that effective learning is influenced by a cycle of experimentation, reflection, research, and exercising’ (Huerta‐Wong & Schoech, 2010, p. 86), elements of which feature in the body of work comprising this review. The experimentation component is well defined and clearly identifiable. Keefe (1979) describes highly structured role‐play situations occurring within an experiential learning component. Similarly, in the live supervision intervention reported by Pecukonis et al. (2016) and Greeno et al. (2017), experiential learning opportunities are described as occurring within a small group format, using a one‐way mirror in a classroom setting to practice with standardised clients. VanCleave (2007) appears to draw on both concepts of experiential learning outlined above: the ‘homework experientials’ featuring in the training intervention comprise a series of practical tasks based on a range of different learning styles, which students complete between sessions to augment the development of empathy. In Ouellette et al.'s (2006) study, reference is made to the importance of adult learning principles and effective active learning paradigms in technology‐supported instructional environments.

Bandura's propositions are also evident within the included studies. VanCleave (2007) draws on social learning theory (Bandura, 1971), recognising that the modelling of skills is important for learning. Ideas about self‐reinforcement (Bandura, 1976) influenced Laughlin (1978), in a consideration of the impact of internal and external motivation. The exploration into the role of self‐efficacy by Rawlings (2008) in skill development was informed by self‐efficacy and social cognitive theory (Bandura, 1997). Behaviour, according to social cognitive theory, is influenced by goals, outcome expectations, self‐efficacy expectations and socio‐structural determinants (Bandura, 1982). Much of the literature indicates the potential impact of students’ self‐efficacy beliefs for the teaching and learning of communication skills in social work education.

Irrespective of which conceptualisation is used, the value of experiential learning has withstood the test of time and is the front runner in terms of the theoretical orientation underpinning the teaching and learning of CST, or specific components of it, both of which are addressed in this review. Toseland and Spielberg (1982) consider experiential learning fundamental to the systematic training that the teaching of communication skills requires. In a review of practice of teaching and learning of communication skills in social work education in England, Dinham (2006) identified a strong emphasis on experiential and participative teaching and learning methods.

Other theories, for example ego psychology in Hettinga (1978) are discussed particularly in the dissertation theses; however, the theoretical orientations underpinning the pedagogical approaches are largely ill‐defined or absent from the outcome studies in this review.

###### Delivery and approach

The included studies do provide some insight into the delivery format and teaching methods under investigation, especially where studies compare teaching modalities or approaches. A concerning issue in the earlier studies is whether practicing skills in communication and empathy (utilising an experiential component) is more effective than a purely didactic traditional lecture‐based approach. Larsen and Hepworth (1978) compared the efficacy of a traditional didactic intervention with an experiential intervention used within communication laboratories. Collins (1984) also compared a lecture‐based training course with a skills lab training course. The results of these studies supported practice‐based experiential learning. By contrast, when Keefe (1979) compared an experiential‐didactic course to a structured meditation experience with a control group, the experiential group did not make the expected gains, whereas those receiving  meditation did. In an extension of the basic design, Keefe (1979) found a combination of experiential training and structured meditation proved most effective.

Some of the more current studies focussed on classroom‐based teaching versus online delivery, an issue particularly relevant in the current global pandemic, which in many instances has seen teaching move to purely online or blended delivery. Ouellette et al. (2006) compared a classroom‐based instructional approach with an online web‐based instructional approach and found no significant differences between the two. In the study reported by Greeno et al. (2017) and Pecukonis et al. (2016) however, live supervision with standardised clients compared favourably with the TAU, which they describe as being online self‐study.

Other studies compared more specific components within the intervention. The role of active learning for students was important whether that included participation in role‐play with peers or simulated clients. Wells (1976) in comparing the use of roleplay with using participants’ own problems, found neither one proved preferential but identified the active experimentation of students as being the key factor in their interpersonal skills development.

The role of the instructor was also an issue of interest. Hettinga (1978) examined the benefits of 1:1 instructor feedback compared with small group feedback, Laughlin (1978) focused on the role of instructor feedback versus self‐evaluation whilst Greeno et al. (2017) and Pecukonis et al. (2016) expressed optimism for the use of live supervision. Again, whilst no claim can be made for whom the feedback provider (self, peers or instructor) should be, active engagement with the evaluation and feedback process seems to be the underlying mechanism which facilitates change. Opportunities for playback was another area for investigation. Reflecting the rapid development of technology in recent years, Laughlin (1978) investigated the use of audiotapes whereas Vinton and Harrington's (1994) instructional package consisted of watching videotapes of themselves or others engaging in communicative interactions. Opportunities to observe practice have a facilitative quality, a point recognised by the study authors who drew on Bandura's work.

Although there are not enough studies comparing like for like to draw any firm conclusions, the current body of research indicates that the rehearsal of skills through role‐play or simulation accompanied by opportunities for observation, feedback and reflection offer benefits for systematic CST, facilitating small gains, on skill‐based outcome measures at least. Some of the authors included in this review are confident in recommending specific teaching methods. Toseland and Spielberg (1982) suggest practice, feedback and modelling are necessary; Schinke et al. (1978) add role playing, cueing, and positive reinforcement to this list. Greeno et al.'s (2017) advice to educators is similar, with the added recommendation of supervision. Pecukonis et al. (2016) highlighted modelling of techniques to students as key. In a review of empathy training in which meta‐analysis was feasible, Teding van Berkhout and Malouff (2015) suggest that studies in which behavioural skills were developed through instruction, modelling, practice and feedback had higher, but not significantly higher, effect sizes than those in which some or all of these components were missing. Findings from qualitative research indicate that students learn communication and interviewing skills through the practice, observation, feedback and reflection that accompany simulation and role‐play activities, which Banach et al. (2020) found mapped onto Kolb's (1984) model of experiential learning. Further exploration of these issues is required.

###### Implementation factors: Amount, duration and uptake

Considerable variation in terms of amount and duration is evident across the included studies. The briefest intervention was a single 4‐h training session (Schinke et al., 1978) whilst the longest intervention, described as ‘extensive’ appears to be interspersed throughout a 4‐year degree course (Barber, 1988). Literature has documented the ability to teach empathy at a minimally facilitative level in as few as 10 h (Carkhuff, 1969c; Carkhuff & Berenson, 1976; Truax & Carkhuff, 1967). Indeed, Larsen and Hepworth (1978) found positive change occurred from a 10‐h intervention, but ‘estimated that 20 h, preferably 2 h per week for 10 weeks, would be ample’ (p. 79). However, Toseland and Spielberg (1982) suggested that the course under investigation in their study, which lasted approximately 45 h (30 h of which were experiential learning in a laboratory) may not be sufficient to increase students’ skill to the level of competence expected of a professional worker. In the study undertaken by VanCleave (2007), implementation of the intervention appeared to vary between students, because ‘when assignment by cohort could not be achieved, training was subdivided into smaller groups. Given the flexibility of the researcher, individual training was accommodated’ (p. 119). It is likely this variation occurred to enhance student participation in the study, maximising data collection opportunities for research purposes.

A number of studies did not report details regarding the amount and duration of the intervention, and some provided rather vague or imprecise details, rendering comparative aims regarding amount and duration of training futile.

The studies focus on what was taught, but data on uptake is sorely lacking. Some of the included studies (Collins, 1984; Larsen & Hepworth, 1978; Ouellette et al., 2006) compared students’ personal and demographic characteristics alongside their pre‐course training and/or experience. The role of sex, age and pre‐course experience were key considerations. Social work courses attract few men compared to women, and often have small cohorts, making judgements on demographic characteristics difficult. Vinton and Harrington (1994), who examined the impact of sex on students’ empathy levels, found women had higher QMEE scores than men at both pre and post‐test. This is consistent with a study undertaken by Zaleski (2016) which found female students in medicine, dentistry, nursing, pharmacy, veterinary, and law were found to have higher levels of empathy than their male peers.

Counterintuitively, age was not found to be significantly correlated to communication skills. Ouellette et al. (2006) queried whether age was a factor in learning, yet summary statements was the only item on their interview rating scale found to be significantly correlated to age. Collins (1984) found that the amount of prior training had no impact on students’ ability to demonstrate interpersonal skills. Similarly, in a comparison of the mean levels achieved by groups dichotomised on the basis of age, sex, previous social work experience, and undergraduate social welfare or other major, Larsen and Hepworth (1978) found such attributes yielded no significant differences on either pre‐ or post‐test scores. Both studies challenge the assumption that students with more social care experience before training possess more or better communication skills than those without. In terms of uptake, Larsen and Hepworth (1978, p. 78) suggested that ‘a mix with contrasting skill levels appears advantageous’, because ‘students with higher‐level skills modelled facilitative responses in the practice sessions for students with lower skills, thus encouraging and assisting the latter to achieve higher levels of responding’. In the study conducted by Laughlin (1978), self‐instruction students exhibited significantly higher mean scores for enjoyment and number of optional practice items completed than students in an instructor‐led group. Self‐instruction ‘creates a sense of self‐reliance, confidence, and personal responsibility for learning which promotes enjoyment and devotion to task not present under circumstances of external control’ (Laughlin, 1978, p. 67). Self‐instruction appears to facilitate uptake. Other issues affecting student learning such as concentration or care‐giving responsibilities and their impact on uptake were not addressed in any of the studies included in this review.

#### 5.2.2 Excluded studies

There were 33 papers covering 30 studies, which narrowly missed the inclusion criteria, or which content experts might expect to see in the review. There were two main reasons for exclusion, both of which are outlined in the review protocol (Reith‐Hall & Montgomery, 2019). First, the study design did not meet the minimum standards of methodological rigour, predominantly because an appropriate comparator was lacking. Second, the population was too specific, drawn from social work courses purely focusing on child welfare or working with children, or too general‐including students drawn from a variety of different courses. A full list of excluded studies and reasons for exclusion is presented in Table 2.

**Table 2:** Excluded studies table.

Author (first)	Date	Reason for exclusion
Andrews	2017	No comparator
Bakx	2006	No comparator
Barclay	2012	No comparator
Bogo	2017	No intervention
Bolger	2014	No comparator
Carrillo	1993	No comparator
Carrillo	1994	Unsuitable comparator
Carter	2018	No comparator
Cartney	2006	No comparator
Cetingok	1988	Insufficient time points
Collins	1987	Unsuitable intervention and comparator
Corcoran	2019	No comparator
Domakin	2013	No comparator
Gockel	2014	No comparator
Hansen	2002	No comparator
Hodorowicz	2018	Population too specific (child welfare training)
Hodorowicz	2020	Population too specific (child welfare training)
Hohman	2015	No comparator
Kopp	1982	No comparator
Kopp^a^	1985	No comparator
Kopp	1990	No comparator
Koprowska	2010	Unsuitable comparator
Lefevre	2010	No comparator
Magill	1985	No comparator
Mishna	2013	Unsuitable comparator
Nerdrum	1995	Population too specific (child care pedagogues)
Nerdrum^a^	1997	Population too specific (child care pedagogues)
Nerdrum^a^	2003	Population too specific (child care pedagogues)
Patton	2020	Population too general (psychology & social justice)
Rogers	2009	No comparator
Scannapieco	2000	Population too specific (child welfare training)
Tompsett	2017	Instrument development
Wodarski	1988	No clear intervention

^a^ Multiple papers of a study already accounted for.

### 5.3 Risk of bias in included studies

Both review authors assessed the risk of bias of the included studies, independently applying the ‘Risk of bias’ tools—ROB 2 (Sterne et al., 2019) for the randomised trials and Robins‐I for the non‐randomised studies of interventions (Sterne, Hernán, et al., 2016). Both tools comprise a set of bias domains, intended to cover all issues that might lead to a risk of bias (Boutron et al., 2021). We used the Methodological Expectations of Cochrane Intervention Reviews (MECIR) guidance (Higgins et al., 2021), The Revised Cochrane risk‐of‐bias tool for randomised trials (RoB 2) (Higgins et al., 2019) and the Risk of Bias in Non‐randomised Studies of Interventions (ROBINS‐I): detailed guidance (Sterne, Higgins, et al., 2016) to inform our judgements. To answer the review's research question, we were interested in assessing the effect of assignment to the intervention, as opposed to adherence to the intervention. Discrepancies between review author judgements were resolved through discussion.

Both reviewers judged there to be a moderate or high/serious risk of bias in all but three of 15 included studies, with only one study receiving a low risk of bias rating overall, with an additional two studies receiving a low bias rating overall for one outcome measure but not the other. The lack of information for certain domains was a problem in all of the studies, highlighting that in future, researchers should report a greater level of detail to enable the risk of bias to be fully assessed. Using a tool such as CONSORT SPI (Grant et al., 2018) would facilitate this.

#### 5.3.1 Risk of bias in randomised trials

As shown in Table 3, there was considerable variation within the risk of bias domains of the non‐randomised studies. Only one study was rated as low risk of bias, one was rated as having ‘some concerns’, three were rated as being at high risk of bias and one study (reported in two papers) received a mix of overall bias ratings, according to the outcomes measured. Limitations were evident in all of the studies, including the lack of information reported in domains 2 and 5.

**Table 3:** Risk of bias summary table for randomised studies based on ROB 2.

Study	Domain 1	Domain 2	Domain 3	Domain 4	Domain 5	Overall risk of bias
	Risk of bias arising from the randomisation process	Risk of bias due to deviations from the intended interventions	Risk of bias due to missing outcome data	Risk of bias in measurement of the outcome	Risk of bias in selection of the reported result	
Hettinga (1978)	LOW	Not reported	HIGH	Self‐perceived skills	Not reported	HIGH
				HIGH		
				Self‐esteem		
				SOME		
Larsen and Hepworth (1978)	LOW	Not reported	LOW	LOW^a^	Not reported	LOW
Laughlin (1978)	LOW	Not reported	HIGH	HIGH	Not reported	HIGH
Greeno et al. (2017)	LOW^b^	Not reported	LOW	Perceived empathy	Not reported	Perceived empathy
				SOME		SOME
				Behaviour change		Behaviour change
				LOW		LOW
Pecukonis et al. (2016)				Self‐efficacy		Self‐efficacy
				SOME		SOME
				Behaviour change		Behaviour change
				LOW		LOW
Schinke et al. (1978)	SOME	Not reported	LOW	Self‐perceived skills	Not reported	SOME
				SOME		
				Behaviour change		
				LOW		
Wells (1976)	SOME	HIGH	HIGH	LOW	Not reported	HIGH

^a^ Information about independent outcome assessors not reported in Larsen and Hepworth's (1978) study was provided in Larsen's (1975) PhD thesis, allowing the risk of bias in Domain 4 to be downgraded from high to low.

^b^ Greeno et al. (2017) and Pecukonis et al. (2016) used the same data set. Email contact with Elizabeth Greeno confirmed that randomisation occurred using SPSS.

##### Domain 1—Bias arising from the randomisation process

Randomisation aims to avoid an influence of either known or unknown prognostic factors. There was considerable variation provided by the study authors regarding the randomisation process. Where there was sufficient information about the method of recruitment and allocation to suggest the groups were comparable with respect to prognostic factors (Hettinga, 1978; Larsen & Hepworth, 1978; Laughlin, 1978), the risk of bias was considered low. This level of detail is provided by Laughlin (1978): a table of random numbers ensured allocation sequence generation; manila envelopes were used for allocation sequence concealment; and potential prognostic factors such as age, prior job and training experience were measured as equivalent for all groups at the outset.

Conversely, information required for ROB 2 was missing from the other studies, some of which was gleaned by directly contacting study authors. Elizabeth Greeno provided additional details about the randomisation process, enabling the risk of bias in the study reported by Greeno et al. (2017) and Pecukonis et al. (2016) to be rated as low. Schinke et al. (1978) and Wells (1976) stated that students were randomly assigned to groups, however they did not provide any details about how students were recruited or allocated. Both authors have passed away so further information could not be ascertained. Although there were no obvious baseline differences between groups to indicate a problem with the randomisation process, the absence of detailed information led to a judgement of some concern for both studies in this domain.

##### Domain 2—Risk of bias due to deviations from the intended interventions (effect of assignment to intervention)

Given placebos and sham interventions are generally not feasible in educational interventions, students and staff tended to be aware of which intervention the students were assigned to, particularly since students were largely drawn from cohorts known to each other. Control group scores were markedly different from intervention scores, suggesting contamination between groups did not occur. In reviewing the papers, there were no reports of control groups receiving the active intervention, nor did trialists report that they had changed the intervention. However, a lack of information about deviations from the intended interventions is reflected in our use of the term ‘not reported’.

Similarly, there was no information as to whether an appropriate analysis had been used to estimate the effect of assignment to intervention. Higgins et al. (2019, p. 26) acknowledge that ‘exclusions are often poorly reported, particularly in the pre‐CONSORT era before 1996’. Apart from the study reported by Pecukonis et al. (2016) and Greeno et al. (2017), the randomised trials included in this review were conducted in the 1970s, which helps to explain why making interpretations of the risk of bias for these empirical studies was particularly difficult. For most of the randomised trials, there was nothing to suggest that there was potential for a substantial impact (on the result) of the failure to analyse participants in the group to which they were randomised. However, again a lack of information led the reviewers to replace a bias rating with ‘not reported’. Wells (1976) study provides an exception to this rule. Noting that two students from each group swapped due to placement clashes, Wells did not perceive this as an issue. However, the data of these students were analysed in terms of the interventions they received rather than the interventions to which they were initially assigned. As a result, both review authors deemed the risk of bias rating to be high for this domain.

##### Domain 3: Risk of bias due to missing outcome data

Some studies (Greeno et al., 2017; Larsen & Hepworth, 1978; Pecukonis et al., 2016; and Schinke et al., 1978) retained almost all of their participants hence no data or very little data were missing, warranting a low risk of bias rating for the missing outcome data domain. Pecukonis et al. (2016) for example, identify low attrition as a strength in their study, highlighting that retention at T3 and T4 was 96% and 94%, respectively (p. 501).

Three studies were judged to be at high risk of bias due to missing data and a lack of any accompanying information. Laughlin (1978) identified that out of 68 students in her study, ‘seven subjects failed to complete either the pre‐ or post‐test because of absence from class on the day these tests were administered’ (p. 40). Information about the group for which data were missing was not provided. In Wells' (1976) study, the four students who were not present at post‐testing were excluded from the analysis, and whilst the number may seem small, they represent a significant proportion of the original study sample, which comprised only 14 students. Hettinga (1978, p. 57) ‘assumes that no interaction of selection and mortality occurred’, yet researcher assumptions do not constitute evidence. In all three of these studies, the reasons for the absences were unclear and there was no evidence to indicate that the result was not biased by missing outcome data. The authors did not discuss whether missingness depended on, or was likely to depend on, its true value. Yet it is possible, likely even, that missingness in the outcome data could be related to the outcome's true value if, for example, students who perceived their communication skills to be poor decided not to attend the post‐test measurements. As a result of this, and the study authors’ lack of attention to these issues, we judged there to be a high risk of bias due to missing outcome data in the trials undertaken by Hettinga (1978), Laughlin (1978), and Wells (1976).

##### Domain 4: Risk of bias in measurement of the outcome

Randomised trials are judged as low risk of bias in measurement of the outcome if: the methods are deemed appropriate, do not differ between intervention groups, and ensure that independent assessors are blinded to intervention assignment. Wells' (1976) study explicitly met this criterion. Based on Larsen and Hepworth's (1978) article, the risk of bias would have been rated conservatively high because the study does not say if the outcome assessors knew to which group the students belonged. However, in her PhD thesis, on which Larsen and Hepworth's (1978) article is based, Larsen (1975) clearly states that three social work raters were blind to the identification of the student and to their intervention/control group status. The additional information enabled reviewers to judge this domain as being at low risk of bias.

In studies where two different outcome measures were used, bias ratings were judged separately, indicated by the split outcomes in domain 4 in Table 3. For Greeno et al. (2017), Pecukonis et al. (2016) and Schinke et al. (1978), low bias ratings were given for measures of behaviour change due to evidence of independent raters, blind to the intervention status of participants. However, the self‐report measures used by each, warrant a higher risk of bias. According to the Rob 2 guidance, for self‐reported outcomes, the assessment of outcome is potentially influenced by knowledge of the intervention received, leading to a judgement of at least some concerns (Higgins et al., 2019, p. 51). If review authors judge it likely that participants’ reporting of the outcome was influenced by knowledge of the intervention received, then a high risk of bias is justified. The adapted Counselor Effectiveness Scale, used by Schinke et al. (1978) required participants to rate their attitudes towards their own performance. In this study, students were aware of which intervention group they belonged to, yet the waiting list control procedure reduced potential issues such as social desirability, hence a rating of some concerns was considered appropriate. In the study reported by Greeno et al. (2017) and Pecukonis et al. (2016), whose subjective measures included perceived empathy and self‐efficacy respectively, it seems probable that students were aware of the intervention group they belonged to. Given there were no differences between groups on either outcome measure, it seems unlikely that participants’ reporting of the outcome(s) was influenced by knowledge of the intervention received. The ‘some concerns’ rating was applied to both.

Hettinga (1978) reports that the researcher had no knowledge as to which treatment groups the participants were randomly assigned. However, the outcome assessors were the students who were completing two subjective measures—the Rosenberg Self‐Esteem Scale and the self‐perceived interviewing competence (SPIC) questionnaire. It is likely that the students were aware of which intervention they received. The lack of change for self‐esteem meant this outcome measure was given the ‘some concerns’ rating. However, we took a more cautious approach to students’ self‐perceived interviewing competence as the results were significant. Knowledge of the intervention could have had an impact, for example, if those students in the self‐instruction group had tried harder. There was no information to determine the likelihood that assessment of the outcome was influenced by knowledge of the intervention received, which led to a conservative judgement from the reviewers of a high risk of bias for this outcome measure.

In the study conducted by Laughlin (1978), the high risk of bias is due to known differences in the measurement of the outcome between the intervention groups. Students in the self‐reinforcement group rated their own empathic responses, whereas the supervisor rated the responses of students receiving the other experimental condition. Higgins et al. (2019, p. 50) point out that, ‘outcomes should be measured or ascertained using a method that is comparable across intervention groups’, which is clearly not the case in this study.

##### Domain 5: Risk of bias in selection of the reported result

Bias due to selective reporting can occur when all the planned results are not completely reported. Whilst there were no unusual reporting practices identified within the randomised studies, none of them had stated their intentions in a published protocol, or additional sources of information in the public domain, making decisions about the risk of bias in selection of the reported result very difficult to ascertain. Greeno et al. (2017) and Pecukonis et al. (2016) report on the same study, hence these papers were compared for consistency, however, they report on different outcomes, limiting the usefulness of this approach. Email contact with Elizabeth Greeno suggests that whilst the authors had a formal plan to follow, this was not published. Consequently, verifying how reported results were selected was not possible. Due to a lack of information in all the included randomised trials, we could not make a risk of bias judgement for this domain.

##### Overall risk of bias

Only one included study (Larsen & Hepworth, 1978) received a low risk of bias rating overall; one study (Schinke et al., 1978) was considered to have some concerns; three studies (Hettinga, 1978; Laughlin, 1978; Wells, 1976) received high risk of bias ratings overall and one study (reported by Greeno et al. (2017) and Pecukonis et al. (2016) varied between low risk and some concerns of risk of bias depending on the outcome measure reviewed. The lack of information, evident in all of the domains is problematic and may have elevated the risk of bias for some studies and in some domains. The absence of protocols or accompanying documentation for the studies has compounded this issue. Boutron et al. (2021) state that the completeness of reporting of published articles is generally poor, and that information fundamental for assessing the risk of bias is commonly missing. Whilst reporting is seen to be improving over time, the majority of the included trials were conducted in the 1970s, and are evidently, a product of their time. Where study authors have not provided sufficient information, we have indicated that information was not reported. We also acknowledge that we adopted a conservative approach, therefore we might have judged the risk of bias harshly, potentially elevating the risk of bias either at the domain level or in the overall bias judgement for some studies. Frequent discussions supported our endeavours to be consistent.

#### 5.3.2 Risk of bias in non‐randomised studies

As shown in Table 4, there are clear similarities across some domains as well as some marked differences in the risk of bias ratings of the non‐randomised studies, which were judged in accordance with Robins‐I. For the overall bias ratings, the review authors either judged there to be a ‘moderate’ or ‘serious’ risk of bias in each study outcome reviewed, or in one instance, a ‘no information’ rating was issued, because assessing the risk of bias was not feasible.

**Table 4:** Risk of bias table for non‐randomised studies based on Robins‐I.

Study	Domain 1	Domain 2	Domain 3	Domain 4	Domain 5	Domain 6	Domain 7	Overall risk of bias
	Risk of bias due to confounding	Risk of bias in the selection of participants	Risk of bias in the classification of interventions	Risk of bias in the deviation of interventions	Risk of bias due to missing outcome data	Risk of bias in measurement of the outcome	Risk of bias in selection of the reported result	
Barber (1988) **(Experiment 1)**	SERIOUS	LOW	LOW	No information	No information	No information	No information	SERIOUS
Barber (1988) **(Experiment 2)**	SERIOUS	LOW	LOW	No information	No information	No information	No information	SERIOUS
Collins (1984)	MODERATE	LOW	SERIOUS	No information	LOW	Analogue measure MODERATE	No information	SERIOUS
						Other measures LOW		
Keefe (1979)	No information	LOW	LOW	No information	LOW	SERIOUS	No information	SERIOUS
Ouellette (2006)	MODERATE	LOW	LOW	No information	LOW	LOW	No information	MODERATE
Rawlings (2008)	MODERATE	LOW	LOW	No information	SERIOUS	Direct practice LOW	No information	SERIOUS
						Self‐efficacy MODERATE		
Toseland (1982)	MODERATE	LOW	LOW	No information	LOW	No information	No information	MODERATE
VanCleave (2007)	MODERATE	LOW	LOW	No information	LOW	Empathic response LOW	No information	Empathic response MODERATE
						Empathic concern SERIOUS		Empathic concern SERIOUS
Vinton (1994)	No information	LOW	LOW	No information	Emotional empathy LOW	Emotional empathy SERIOUS	No information	Emotional empathy SERIOUS
					Expressed empathy No information	Expressed empathy No information		Expressed empathy No information

##### Domain 1: Bias due to confounding

Sterne, Higgins, et al. (2016, p. 20) suggest ‘baseline confounding is likely to be an issue in most or all NRSI’, which was reflected in the included studies of this review. The lack of information in two of the studies (Keefe, 1979; Vinton & Harrington, 1994) meant that an assessment of bias of confounding could not be provided. The other non‐randomised studies were rated as having at least moderate risks of confounding, since by the nature of their designs, causal attribution was not possible. As one study author comments, ‘selection of a nonrandom design subjected the research to confounds and threats to validity’ (VanCleave, 2007, p. 105). Indeed, VanCleave (2007) discusses optimal group equivalency, and suggests that the distributions of some key confounders ‘fell pretty evenly’ (p. 135) between the intervention and control groups hence a moderate risk of bias was appropriate.

Whilst it is clearly not possible to control for all confounders, attempts were made by some study authors to use an analysis method that controlled for some of the most obvious ones, resulting in judgements of moderate risk of bias. Collins (1984) measured pre‐existing group differences, analysed them using a *χ* ^2^ test and found them to be unproblematic. Toseland and Spielberg (1982) used *χ* ^2^ and Kendall's *T* to measure a wide range of confounding variables, such as age, educational experiences and previous human services experience, from which they determined that students in the intervention and control groups were similar to one another regarding key characteristics. Ouellette et al. (2006) performed similar analyses on a wider range of confounding variables, which included age, credit hours and hours per week of paid employment undertaken during the semester, previous interviewing experience, grade point average and paid employment hours. Age was the only variable to be statistically significant; the online group were a little older than the classroom group.

In a design comparing first and final year students, Rawlings (2008) sought to establish comparability betwixt groups based on sex, ethnicity, grade point average, and age. Again, it appeared only age was significant, reflecting the fact that the final year students were further into their studies than those entering their first year. Barber (1988) employed a similar design, however both experiments were rated as having a serious risk of bias due to confounding factors. Student characteristics were not measured in either experiment, so it is impossible to be sure that the group receiving the microskills training did not differ in some way (other than the dependent variable) to the comparator student cohort.

##### Domain 2: Bias in selection of participants into the studies

This domain is only concerned with ‘selection into the study based on participant characteristics observed *after* the start of intervention… the result is at risk of selection bias if selection into the study is related to both the intervention and the outcome (Sterne, Higgins, et al., 2016, p. 30). There was nothing to suggest that any students were selected based on participant characteristics after the intervention had commenced in any of the studies, therefore a low risk of bias was given to all of the studies for this domain.

##### Domain 3: Bias in classification of interventions

All of the non‐randomised studies used population‐level interventions therefore the population is likely to be clearly defined and the collection of the information is likely to have occurred at the time of the intervention (Sterne, Higgins, et al., 2016, p. 33). As a result, the bias ratings for this domain were low in almost all of the studies. We could have issued no information ratings but decided a low rating was probably a better reflection of the non‐randomised studies in this domain. One study provides an exception to the rule. Collins (1984, p. 67) stated, ‘it was not possible to establish a control group where no laboratory training took place’. This suggests the lecture‐trained and lab‐trained groups were not as distinctly different as was necessary, hence the serious risk of bias rating was applied for this domain.

##### Domain 4: Bias due to deviations from intended interventions

None of the studies reported on whether deviation from the intended intervention took place, hence the no information rating was issued for this domain across all of the studies.

##### Domain 5: Bias due to missing data

For some of the non‐randomised studies (Collins, 1984; Keefe, 1979; Ouellette et al., 2006; Toseland & Spielberg, 1982), data sets appeared complete or almost complete. In VanCleave's (2007) study, where attrition was slightly higher, the number of missing participants was similar across the intervention group (*N* = 3) and control group (*N* = 2); reasons for drop‐out were also provided. A low bias rating was given for the missing data domain in these studies.

In Vinton and Harrington's (1994) study, a complete data set was provided for the QMEE scores, hence a low bias rating judgement was warranted, but the absence of student numbers for the Carkhuff scores meant a bias rating for this outcome measure could not be issued. An absence of information, on which to base a judgement, was also reflected in the results of Barber's (1988) experiments.

In Rawlings' (2008) study, results were reported as if all student data were present, however data were missing for some of the entering students. It is concerning that the results tables do not acknowledge the missing data. An imputational approach such as last observation carried forward or the use of group means would have enabled missing data to be dealt with, but instead the researcher has simply analysed the data available. Given that the missingness is not explained, both reviewers agreed that a serious risk of bias was justified.

##### Domain 6: Bias in measurements of outcomes

The timing of outcome measurements was problematic in three of the studies. A delay of approximately 3 weeks occurred in Collins' (1984) study for students completing the analogue measures, which reduced the time gap between pre‐and‐post‐test training scores. A bias rating of moderate concern was justified given this could have led to an under‐estimation of the positive gains made by students on this outcome measure.

In Keefe's (1979) study, although students were tested after their respective interventions, the interventions were of different durations hence the data collection time points varied. These are not comparable assessment methods. The meditation group was also tested three times, thus familiarity with the test may have produced the higher scores on the Affective Sensitivity Scale, rather than demonstrating a genuine improvement. Keefe (1979) states that levels of meditation attainment were blind rated (p. 36), however students in the experiential intervention group self‐assessed only, the subjectivity of which increased bias in the measurements of outcomes. These issues elevated the risk of bias in this domain to serious.

VanCleave (2007) reports, ‘the Davis self‐inventory was completed by the participant before, or following, each 8 excerpt role played situation’ (p. 118). Inconsistency surrounding the timing of when the instrument was completed led to a serious bias rating for the outcome measure of empathic concern and perspective taking. However, a low rating was given for empathic response where timing issues were not a cause for concern and independent raters were not aware of students’ intervention group status. The different ratings applied to each outcome is represented by the split ratings for this domain in Table 4.

The same approach of splitting the outcome measures domain was taken in Rawlings' (2008) study. The direct practice outcome was judged to have a low risk of bias rating because assessors were blinded to the intervention status, whereas the self‐efficacy outcome received a moderate risk of bias rating, as the students themselves were the outcome assessors. Given the students comprised discreet cohorts, knowledge of the intervention group was not considered problematic by the reviewers. Conversely, the self‐assessment measure in Vinton and Harrington's (1994) study warranted a serious risk of bias rating. The potential for study participants to be influenced by knowledge of the intervention they received was considerable. The emotional empathy scores of the control group dropped considerably at post‐test, which could be an indication that the students had become aware that their peers were receiving beneficial interventions aimed at developing empathy, which they were not. Discussions between students were more likely in this study given they were all in the same cohort. Contamination effects could have impacted students’ self‐assessment scores.

Independent outcome assessors and appropriate blinding were used in all of the outcome measures used in Collins' (1984) study and in the video‐tape interviews in Ouellette et al.'s (2006) study, which, with the exception of the timing issues associated with Collins' (1984) analogue measure, resulted in low bias ratings for the outcomes measures in these two studies.

Key information was lacking in some studies. Notably in Barber's (1988) experiments, a judgement about the methods of outcome assessment could not be made at all due to the absence of information. Toseland and Spielberg (1982) described their judges as being independent but did not state whether or not they were aware of which intervention the student had received. For the outcome relating to empathic response, Vinton and Harrington (1994) provided no information about blinding or the independence of the outcome assessors. Potentially then, this study is also at risk of researcher allegiance bias. If, for example, the outcome assessors were part of the same institution as the instructors and the students, or of even more concern, if the assessors *were* the instructors, then this could pose a serious risk of bias, because potentially they have a vested interest in the findings. It was not possible to establish assessor independence, so the reviewers opted for a ‘no information’ rating for the Carkhuff scales outcome measurement in Vinton and Harrington's (1994) study.

Research suggests that if study authors play a direct role, studies are more likely to be biased in favour of the treatment intervention (Eisner, 2009; Maynard et al., 2017; Montgomery & Belle Weisman, 2021). There is a distinct possibility that researchers of the included studies delivered the interventions themselves, leading to a further source of bias. VanCleave, for example, who had 19 years of teaching experience as an adjunct in the university where her research was conducted, acknowledged that ‘the researcher acted as teacher and facilitator in the intervention, which is typically not a recommended research strategy’ (VanCleave, 2007, p. 117). The same issue is likely present in at least some of the other non‐randomised studies, although there was a lack of information from which to establish its presence or impact.

##### Domain 7: Bias in selection of reported results

There was no obvious bias in the reporting of results for any of the reported outcomes in the non‐randomised studies, however, there were no protocols or a priori analysis plans with which to compare the reported outcomes with the intended outcomes. Studies were not reported elsewhere hence external consistency could not be established. The ‘no information’ category was deemed most appropriate by both reviewers.

##### Overall risk of bias judgement

Only two studies (Ouellette et al., 2006; Toseland & Spielberg, 1982) received an overall bias rating of moderate, reflecting a moderate rating in the confounding domain. Other studies (Barber, 1988; Collins, 1984; Keefe, 1979; Rawlings, 2008) were considered to be at serious risk of bias overall, due to receiving a serious risk of bias rating in at least one domain. For one study (Vinton & Harrington, 1994), the absence of information in several domains led to a ‘No information’ rating in the overall risk of bias judgement for one outcome measure but a serious risk of bias in another. Similarly, another study (VanCleave, 2007) also received a split rating for the overall risk of bias domain, with a moderate risk of bias for one outcome measure and a serious risk of bias for the other.

### 5.4 Effects of interventions

The results, as shown in Table 5, are reported for the data that is available, relevant to answering the research question, using either the mean post‐test differences between intervention groups and control groups or the mean change score between the two groups. As outlined in Section 5.2.1, extreme clinical heterogeneity exists between the included studies of this review, in terms of study designs, population characteristics, intervention types and features, comparators, outcomes and outcome measures. For example, in what appears to be the most promising examples of comparable situations‐empathic understanding‐the heterogeneity of the intervention is too broad to meta‐analyse data in a meaningful way. Of the four studies measuring empathic understanding (Greeno et al., 2017; Keefe, 1979; VanCleave, 2007; Vinton & Harrington, 1994), the intervention types and characteristics, as shown in the included studies table, are vastly different. They range from 2 days of a motivational interviewing intervention consisting of live supervision with standardised clients (Greeno et al., 2017), to 3 months of role‐play and 3 weeks of meditation (Keefe, 1979), to a multitude of components including art and music (VanCleave, 2007) to the use of videotapes of an unspecified amount and time period (Vinton & Harrington, 1994). Meta‐analysing such disparate interventions would therefore not be meaningful.

**Table 5:** Results table of outcomes.

First author, date	Outcome measure	Outcome type	Effect Size and confidence intervals
Barber, 1988	Counselor Rating Form (non‐verbal communication only)	Level 2b—Acquisition of Knowledge	Responsive interviews: Expertness −0.82 (−1.5421 to −0.099) Attractiveness −0.80 (−1.5223 to −0.0818) Trustworthiness −0.82 (−1.5402 to −0.0974) Unresponsive interviews: Expertness −0.84 (−1.5656 to −0.1195) Attractiveness −1.25 (−2.0066 to −0.4916) Trustworthiness −0.87 (−1.5897 to −0.1404)
Barber, 1988	Counselor Rating Form	Level 2b—Acquisition of Knowledge	Responsive interviews: Expertness −2.80 (−3.589 to −2.027) Attractiveness −1.49 (−2.114 to −0.861) Trustworthiness −1.45 (−2.074 to −0.828) Unresponsive interviews: Expertness −1.50 (−2.132 to −0.877) Attractiveness −1.81 (−2.466 to −1.150) Trustworthiness −1.88 (−2.5404 to −1.2102)
Collins, 1984	Skills Acquisition Measure	Level 2b—Acquisition of Skills	Empathy 1.21 (0.566 to 1.844) Warmth 1.37 (0.726 to 2.023) Genuineness 1.77 (1.090 to 2.441)
	Carkhuff stems	Level 2b—Acquisition of Skills	Empathy 0.60 (−0.069 to 1.265) Warmth 0.78 (0.102 to 1.448) Genuineness 1.13 (0.444 to 1.824)
	Analogue	Level 2b—Acquisition of Skills	Empathy 1.74 (1.027 to 2.455) Warmth 1.80 (1.078 to 2.514) Genuineness 1.88 (1.156 to 2.605)
Greeno, 2017	Toronto Empathy Questionnaire (TEQ)	Level 2a—Modification in attitudes and perceptions (Perceived empathy)	−0.26 (−0.798 to 0.274)
	Motivational Interviewing Treatment Integrity (MITI) questionnaire	Level 2b—Acquisition of Skills	0.24 (−0.317 to 0.797)
Pecukonis, 2016	Self‐efficacy scale	Level 2a—Modification in attitudes and perceptions (Self‐efficacy)	Insufficient data to report effect size and confidence intervals
	Motivational Interviewing Treatment Integrity (MITI) questionnaire	Level 2b—Acquisition of Skills	Empathy 0.24 (−0.319 to 0.797) MI spirit 0.12 (−0.434 to 0.680) % MI adherent behaviours 0.34 (−0.225 to 0.896) % Open questions 0.15 (−0.407 to 0.707) % Complex reflections −0.25 (−0.808 to 0.308) Reflection: Question ratio 0.04 (−0.519 to 0.594)
Hettinga, 1978	Rosenberg Self‐Esteem Scale (RSE)	Level 2a—Modification in attitudes and perceptions (Self‐esteem)	Section 1: 0.43 (−0.481 to 1.340) Section 2: −0.86 (−2.001 to 0.2782)
	Self‐Perceived Interviewing Competence (SPIC) Questionnaire	Level 2b—Acquisition of Skills	Section 1: 1.10 (0.131 to 2.062) Section 2: 0.64 (−0.285 to 1.561)
Keefe, 1979	Kagan affective sensitivity scale	Level 2a—Modification in attitudes and perceptions	Experiential training 0.02 (−0.638 to 0.671) Experiential plus meditation 0.32 (−0.3267 to 0.9748)
Larsen, 1978	Index of Therapeutic Communication Carkhuff	Level 2b—Acquisition of Skills	1.51 (1.0366 to 1.9774)
Laughlin, 1978	Carkhuff's Empathy scale	Level 2b—Acquisition of Skills	1.22 (0.4499 to 1.9894)
	Enjoyment question ranked 1 to 5	Level 1—Learner Reactions	Effect size and confidence intervals cannot be calculated from data available
Ouellett, 2006, a	Basic practice interviewing scale	Level 2b—Acquisition of Skills	Total: 0.24 (−0.661 to 1.147) Attentiveness: 0.73 (0.029 to 1.482) Relaxed: 0.93 (0.147 to 1.710)
	Satisfaction with instruction scale	Level 1—Learner Reactions	Learning exercises well organised −0.21 (−0.961 to 0.540) Learning exercises sparked my interest −0.05 (−1.224 to 0.292) I enjoyed participating in learning exercises −0.23 (−0.982 to 0.520) Instructions were clear 0.46 (−2.94 to 1.223)
Rawlings, 2008	Self‐efficacy scale	Level 2a—Modification in attitudes and perceptions (Self‐efficacy)	Beginning 2.50 (1.5753 to 3.425) Exploring 1.30 (0.535 to 2.060) Contracting 2.04 (1.1898 to 2.8999) Case Management 2.16 (1.2896 to 3.0339) Core conditions 1.27 (0.5147 to 2.0348) Total 2.04 (1.1881 to 2.8977)
	Direct practice skills	Level 2b—Acquisition of Skills	Beginning 1.78 (0.9627 to 2.6006) Exploring 1.52 (0.7298 to 2.3022) Contracting 1.69 (0.8862 to 2.5017) Case Management 1.67 (0.8622 to 2.4708) Core conditions 1.28 (0.5177 to 2.0385) Total 1.85 (1.019 to 2.6741)
Schinke, 1978	Counselor effectiveness scale	Level 2a—Modification in attitudes and perceptions	0.93 (0.0682 to 1.7903)
	Videotaped interview ratings	Level 2b—Acquisition of Skills	Eye contact 0.75 (−0.0984 to 1.594) Smiles 0.34 (−0.4834 to 1.1647) Nods 0.93 (0.0684 to 1.7906) Forward trunk lean 1.36 (0.4554 to 2.2715) Open‐ended questions 1.01 (0.1391 to 1.876) Closed‐ended questions −0.24 (−1.0601 to 0.582) Content summarisations 0.98 (0.1124 to 1.8436) Affect summarisations 0.82 (−0.0317 to 1.6719) Incongruent response −0.68 (−1.5221 to 0.1608)
Toseland, 1982	Carkhuff Communication Index	Level 2b—Acquisition of Skills	1.40 (0.7506 to 2.0477)
	Carkhuff Discrimination Index	Level 2b—Acquisition of knowledge	−1.31 (−1.9563 to −0.6694)
	Counselling Skills Evaluation Part 1 (Communication)	Level 2b—Acquisition of Skills	1.20 (0.5588 to 1.8327)
	Counselling Skills Evaluation Part 2 (Discrimination)	Level 2b—Acquisition of Knowledge	−0.53 (−1.1421 to 0.0799)
VanCleave, 2007	Davis’ Interpersonal Reactivity Index (IRI)	Level 2a—Modification in attitudes and perceptions	0.22 (−0.3684 to 0.8041)
	Carkhuff's Index for Communication scripts (CIC)	Level 2b—Acquisition of Skills	1.79 (1.0969 to 2.4799)
Vinton, 1994	Questionnaire Measure of Emotional Empathy (QMEE)	Level 2a—Modification in attitudes and perceptions	0.21 (−0.4536 to 0.8751)
	Carkhuff's empathy scale	Level 2b—Acquisition of Skills	0.88 (0.1823 to 1.5677)
Wells, 1976	A variant of the Carkhuff communication test Carkhuff's empathy scale	Level 2b—Acquisition of Skills	0.84 (−0.4499 to 2.1372)

^a^ Effect size and confidence intervals calculated by study authors.

Gagnier et al. (2013) identified twelve recommendations for investigating clinical heterogeneity in systematic reviews. In terms of the review team, one of us (PM) is a methodologist and the other (ERH) has significant relevant clinical expertise. ERH regularly discussed issues relating to population, intervention and measurement characteristics with the stakeholder group‐who included educators, students and people with lived experience. This provided a range of different perspectives, encouraging us to be reflective and reflexive in our approach, including recognising our own biases. In relation to planning and the rationale for the selection of clinical variables we hoped to consider, these were described a priori in the protocol. Other methods require statistical calculations for which we did not have sufficient data. For example, we had hoped to perform a subgroup analysis relating to the intensity of the interventions, but such data were not sufficiently available‐absent in four of them and described in non‐numerical terms (e.g., as ‘extensive’ or ‘one day’) in a further three. Gagnier et al. (2013) acknowledge the challenge posed by the incomplete reporting of data.

Given the extreme clinical heterogeneity, meta‐analysis was neither feasible nor meaningful. Instead, the findings are synthesised narratively and are organised according to a refined version of a classification of educational outcomes, developed by Kirkpatrick (1967); which is well‐known and widely used. It was refined by Kraiger et al. (1993) to distinguish between cognitive, affective and skill‐based outcomes, and adapted by Barr et al. (2000) followed by Carpenter (2005) for use in social work education. The refined classification comprises: Level 1—Learners’ Reaction, Level 2a—Modification in Attitudes and Perceptions, Level 2b—Acquisition of Knowledge and Skills, Level 3—Changes in Behaviour, Level 4a—Changes in Organisational Practice and Level 4b—Benefits to Users and Carers. Most of the studies reported more than one outcome, but none included level 4 outcomes. Therefore, the findings are synthesised according to an expanded version of levels 1 to 3—learner reactions; attitudes, perceptions, self‐efficacy; knowledge; skills and behaviours.

#### 5.4.1 The importance of empathy

Reported in 9 of the 15 included studies (Collins, 1984; Greeno et al., 2017; Keefe, 1979; Larsen & Hepworth, 1978; Laughlin, 1978; Pecukonis et al., 2016; Toseland & Spielberg, 1982; VanCleave, 2007; Vinton & Harrington, 1994; Wells, 1976), empathy is a common topic of interest within this review. The pivotal role of empathy in social work practice is widely acknowledged (Forrester et al., 2008; Gerdes & Segal, 2009; Lynch et al., 2019), hence the need for students to develop empathic abilities is deemed critical for preparing them for social work practice (Greeno et al., 2017; Zaleski, 2016). As a skill which can be ‘taught, increased, refined, and mediated’ (Gerdes & Segal, 2011, p. 143), it is hardly surprising that empathy features so frequently within the empirical literature. Truax & Carkhuff (1967 (p. 46) describe empathy as ‘the ability to perceive accurately and sensitively the feelings, aspirations, values, beliefs and perceptions of the client, and to communicate fully this understanding to the client’. As study authors Vinton and Harrington (1994, p. 71) point out, ‘these are separate but related phenomenon’. Empathy is a multifaceted phenomenon (Lietz et al., 2011), often conceptualised as empathic understanding and empathic behaviour or response. Empathic understanding consists of cognitive empathy—understanding another person's thoughts or feelings and emotional empathy—the affect invoked by another person's expression of an emotion. Empathic behaviour or response is action‐based—the communicated empathic response, including verbal and non‐verbal communication, to another person's distress (based on accurate cognitive and/or emotional empathy). There is a lack of consensus regarding how empathy should be conceptualised and measured, some of which is reflected within the included studies.

#### 5.4.2 Level 1—Learner reaction outcomes

Learner reactions include students’ satisfaction with the training and their views about the learning experience. As stated in the protocol (Reith‐Hall & Montgomery, 2019), learner satisfaction alone was not sufficient to be regarded as an outcome in this review, and qualitative findings were excluded. Two of the included studies gathered quantitative data on learner reactions, in addition to other outcomes. Laughlin (1978) found self‐instruction students exhibited significantly higher mean scores for enjoyment and number of optional practice items completed than students in an instructor‐led group. Laughlin (1978, p. 67) suggests self‐instruction ‘creates a sense of self‐reliance, confidence, and personal responsibility for learning which promotes enjoyment and devotion to task not present under circumstances of external control’. However, there was no significant correlation between the variables of enjoyment and commitment with students’ gain scores.

Ouellette et al. (2006) issued a semester survey questionnaire, including a four‐item subscale which measured students’ perception of their satisfaction with the instruction they received—traditional classroom based versus online. Most students agreed or strongly agreed that learning exercises were clear and effective, irrespective of the type of instruction they received. There were no significant differences in their satisfaction scores. Again, there was no statistically significant correlation between students’ perceived satisfaction, perceived acquisition of interviewing skills and the independent ratings of students’ acquisition of interviewing skills, in either group.

#### 5.4.3 Level 2a—Modification in attitudes and perceptions

Carpenter (2005, 2011) suggests that Level 2a outcomes relate to changes in attitudes or perceptions towards service users and carers/care‐givers, their problems and needs, circumstances, care and treatment. Motivational outcomes and self‐efficacy also comprise this level (Kraiger et al., 1993).

##### Attitudes and perceptions towards clients

Students’ perceptions towards clients was an outcome of interest for a number of studies included in this review. Affective sensitivity (Keefe, 1979), emotional empathy (Vinton & Harrington, 1994), empathic concern and perspective taking (VanCleave, 2007) and perceived empathy (Greeno et al., 2017) all fit under the umbrella term of empathic understanding. Within the literature, empathic understanding has been further defined as an affective process and a cognitive process. These different ways of conceptualising empathy are evident within the included studies, and in the choice of measuring instruments the researchers employed.

##### Affective and cognitive outcomes

To ascertain students’ abilities to detect and describe the immediate affective state of clients, Keefe (1979) employed Kagan's scale of affective sensitivity (Campbell et al., 1971), which consists of multiple‐choice items used with a series of short, videotaped excerpts from actual counselling sessions. In Keefe's study, a positive and significant effect size of 0.32 was only found once the intervention group had been taught meditation in addition to the experiential training they received, correlating with blind ranked levels of meditation attainment. Keefe (1979) reported that the combined effects of both conditions produced mean empathy levels beyond those attained by master's and doctoral students. Segal et al. (2017, p. 98) suggest that using meditation can promote emotional regulation, which can be considered fundamental to empathy. Dupper (2017, p. 31) suggests that mindfulness is an effective strategy for ‘reducing implicit bias and fostering empathy towards members of stigmatised outgroups’. Both propositions could explain why the combined interventions in Keefe's (1979) study proved most effective.

Also viewing empathy as an affective state, Vinton and Harrington (1994) sought to assess students’ ‘emotional empathy’, which they describe as ‘the ability to be affected by the client's emotional state’ (p. 71). Vinton and Harrington (1994) employed a different outcome measure—the Questionnaire Measure of Emotional Empathy (QMEE) (Mehrabian & Epstein, 1972), which emphasises the affective component of empathy including emotional arousal to others’ distress. Two intervention groups received an instruction package utilising videotapes, one relying on self‐instruction, the other also receiving input from an instructor and peer group, whilst the control group received no intervention. At post‐test, we found a small effect size of 0.21 between the ‘video other and self’ and the controls, however the QMEE scores of both groups had actually declined. Despite these results, Vinton and Harrington (1994) suggested that further investigation into the use of videotape or film is warranted.

Building on the suggestion by Vinton and Harrington (1994) that film can assist the development of empathic understanding, the students in VanCleave's (2007) study watched a 2‐h commercial film, with 30 min of reflection and discussion. The self‐report measure they used comprised two subscales from the Interpersonal Reactivity Index (IRI) (Davis, 1980): the first, empathic concern addresses the affective component of empathy and the second, perspective taking focusses on the cognitive component of empathy. Despite using a broader conceptualisation of empathy and a more inclusive measure, which produced an effect size of 0.22, changes were not statistically significant.

Utilising a different instrument still, Greeno et al. (2017) sought to measure students’ perceived empathy using the Toronto Empathy Questionnaire (TEQ) (Spreng et al., 2009), which views empathy as an emotional process, but is based on items from the QMEE and the IRI. The effect size at post‐test was −0.26, with study authors reporting no statistically significant difference between groups. Given a behavioural measure of empathy used by Greeno et al. (2017) demonstrated a statistically significant small effect size for the intervention group, ‘the lack of change across time and groups’ on the self‐reported TEQ scores was ‘unexpected’ (p. 803).

No statistically significant changes in students’ empathic understanding were identified in the studies above, irrespective of the type of self‐report measure used. The challenges of measuring empathy through self‐reports (Lietz et al., 2011) are clearly evident in this review and will be discussed further in Section 6.

##### Perceptions of the treatment/intervention

Based on the same study reported by Greeno et al. (2017), Pecukonis et al. (2016) issued a 17‐item self‐report measure to garner students’ perceptions of Motivational Interviewing. Training for the intervention group included real‐time feedback by clinical supervisors whereas the control group received online TAU. No between group difference was identified, however perceptions of the Motivational Interviewing increased (by an average of 7 points) for both groups over time.

##### Self‐esteem and self‐efficacy

Self‐esteem, which reflects how people perceive themselves and includes a sense of goodness or worthiness, was an outcome measure in just one of the included studies. Hettinga (1978) argued that self‐esteem, as a critical dimension of professional self‐dependence, directly relates to the attainment of skills. However, he used The Rosenberg Self‐Esteem Scale (RSE) (1965), an instrument measuring *global* self‐esteem, in his study. For students in the intervention group, who experienced videotaped interview playback with instructional feedback, the self‐esteem score dropped very slightly. For the control condition, who received feedback delivered in a small group format, the self‐esteem score remained unchanged. Although we found a small effect size for Section 1, Hettinga suggested the findings were not significant, indicating the intervention had no impact on students’ self‐esteem scores.

Parker (2006) differentiates between the global nature of self‐esteem and the context specific nature of self‐efficacy. Perceived self‐efficacy beliefs ‘influence whether people think pessimistically or optimistically and in ways that are self‐enhancing or self‐hindering’ (Bandura, 2001, p. 10), which has implications for students’ skill development. Self‐efficacy is ‘an individual's assessment of his or her confidence in their ability to execute specific skills in a particular set of circumstances and thereby achieve a successful outcome’ (Bandura, 1986, as quoted in Holden et al., 2002). Literature in the counselling field indicates that self‐efficacy may predict performance (Larson & Daniels, 1998), and can thus serve as a proxy measure. The idea that self‐efficacy is a means to assess outcomes in social work education has gained traction in recent years (Holden et al., 2002, 2017; Quinney & Parker, 2010).

Two of the included studies measured self‐efficacy. Pecukonis et al. (2016) found no change in students’ self‐efficacy scores, either between the brief motivational interviewing intervention group and the TAU control group, or over time. Rawlings (2008), who evaluated the impact of an entire university degree, found students exiting Bachelor of Social Work (BSW) Education had significantly higher self‐efficacy scores (mean score of 6.78) than those entering it (mean score of 4.40). Through multiple regression analysis, results showed that BSW education positively predicted self‐efficacy. However, students’ self‐efficacy ratings did not correlate with their practice skill ratings. Surprisingly, after controlling for BSW education, self‐efficacy was found to be a negative predictor of direct practice skill. Rawlings (2008, p. xi) explains that ‘self‐efficacy acted as a suppressor variable in mediating the relationship between education and skill’. This unexpected finding reflects the controversy surrounding the use of self‐efficacy as an outcome measure, which will be revisited in Section 6.3.

Schinke et al. (1978) asked students to rate their attitudes towards their own role‐played interviewing performance. A large effect size of 0.93 indicates that CST positively affected the attitudes students had about their performance.

#### 5.4.4 Level 2b—Acquisition of knowledge and skills

##### Knowledge

The acquisition of knowledge relates to the concepts, procedures and principles of working with service users and carers. Carpenter (2005), after Kraiger et al. (1993), separated knowledge outcomes into declarative knowledge, procedural knowledge and strategic knowledge. Only procedural knowledge—‘that used in the performance of a task’ (Carpenter, 2011, p. 126), featured as an outcome in this review, reported in three studies (two publications).

###### Procedural knowledge

Barber, 1988 (p. 4) anticipated that students beginning their training would have ‘little knowledge of correct interviewing behaviour’. Conversely, he expected students approaching the end of their training to be more able to judge responsive and unresponsive non‐verbal communication—displayed by actors towards simulated clients (in experiment 1) and practitioners towards real clients (in experiment 2). The anticipated enhanced judgement that microskills training was expected to elicit can be identified as what Kraiger et al. (1993) referred to as procedural knowledge. The experiments used case studies to which students were asked to respond; Carpenter (2011) suggests these are appropriate measures to assess procedural knowledge in social work education.

Contrary to his expectations, and the findings of the other studies in this review, the two experiments conducted by Barber (1988) found that the reactions of students who had received microskills training were less accurate than the reactions of untrained students. In the first experiment, the untrained comparator group rated counsellor responsiveness higher than the trained intervention group, with large effect sizes between the groups for expertness (−0.82), attractiveness (−0.80), and trustworthiness (−0.82). The same pattern emerged when rating counsellor unresponsiveness, with large effect sizes for expertness (−0.84), attractiveness (−1.25) and trustworthiness (−0.87). Flaws in the first experiment include that video segments assessed by students were just 2 min long and included non‐verbal communication only, which goes some way towards explaining the surprising results. Whilst non‐verbal communication is extremely important, the absence of the verbal accompaniment and speech tone, emphasis and pacing, does not reflect how most people communicate, either in their personal lives or in social work practice, nor does it provide students with an opportunity to identify mirroring or mimicry. Barber (1988) acknowledges that artificiality might have led to trained students being more critical than their non‐trained counterparts.

In the second of Barber's experiments, the untrained comparator group rated counsellor responsiveness higher than the trained intervention group, with very large effect sizes between the groups for expertness (−2.80), attractiveness (−1.49) and trustworthiness (−1.45). A similar trend occurred when rating counsellor unresponsiveness with large effect sizes for expertness (−1.50), attractiveness (−1.81) and trustworthiness (−1.88). Barber (1988) found untrained students performed similarly to clients’ ratings, which he perceived as evidence that the trained students were underperforming. However, it is possible that the trained students were looking out for different responses than the untrained students and clients. Barber speculated that training reduced student's capacity to empathise with the client, however, the outcomes of interest—trustworthiness, attractiveness and expertness, which is what students were asked to rate, do not measure empathy, hence the face validity of this measurement is questionable. After completing a factor analysis of a shortened version of the Counsellor Rating Form used in Barber's experiments, Tryon (1987, p. 126) concluded that ‘further information about what it measures, and how, is needed’. It is hard to fathom how the conclusions Barber drew, were born out of the measures he employed and the results these measures produced.

Design limitations are also apparent, with Barber acknowledging that the first year and final year student groups may have been different to each other on variables other than the training. The experiments are important, because the findings that social work students appeared less able to judge responsive and unresponsive interviewing behaviour after training in microskills than counterparts who had yet to receive the training would suggest this teaching intervention could have an adverse, undesirable or harmful effect. However, other studies which ensured that students were matched on factors such as demographic variables and pre‐course experience (e.g., Toseland & Spielberg, 1982), produced more positive results. Thus, Barber's paper is an exception to the rule, such that his findings should be interpreted cautiously, with due consideration of the measurement and design issues evident within both experiments and the serious risk of bias, due to confounding.

In Toseland and Spielberg's (1982) study, two of the four measures employed also tap into the procedural knowledge outcome because students judged the ability of others to respond in a helpful way. First, a film of client vignettes was shown to students who had to select from five different responses, rating them from ‘destructive’ to ‘most helpful’ using the second part of a Counselling Skills Evaluation. Second, through the Carkhuff's Discrimination Index (Carkhuff, 1969a), students rated the helpfulness of four counsellor responses to a set of client statements. Difference scores were generated by comparing students’ ratings with those produced by trained judges. Discrimination scores indicated that students who had received the training were better able to discriminate between effective and ineffective responses to clients’ problems, and their ratings closely matched those of trained judges. With effect sizes of −1.31 for the Carkhuff Discrimination Index and −0.53 for the Counselling Skills Evaluation part 2, and a very high confidence level of 0.001, the findings were significant.

##### Skills

Skills have been organised hierarchically within the literature on social work education outcomes to include initial skill acquisition, skill compilation and skill automaticity (Carpenter, 2005, 2011; Kraiger et al., 1993). Skill automaticity did not feature as an outcome in this review, which possibly reflects the point made by Carpenter (2005); that ‘the measurement of the highest level of skill development, automaticity, poses significant problems’ (p. 14). To our knowledge, no valid measure of automaticity for communication skills currently exists.

###### Initial skills

Initial skills, which are often practised individually, in response to short statements or vignettes, were the most popular outcome reported in this review. ‘Trainee behaviour at the initial skill acquisition stage of development may be characterised as rudimentary in nature’ (Kraiger et al., 1993, p. 316).

The initial skills considered fundamental for demonstrating empathy were evidently interesting to the researchers of the included studies. Variations of the Carkhuff scales (Carkhuff, 1969a, 1969b), which are widely used in social work education (Hepworth et al., 2010), were employed in seven of the included studies (Collins, 1984; Larsen & Hepworth, 1978; Laughlin, 1978; Toseland & Spielberg, 1982; VanCleave, 2007; Vinton & Harrington, 1994; Wells, 1976). The Carkhuff scales comprise two subsets: empathy discrimination (being able to accurately identify the level of empathy response) and empathy communication (putting that discriminated empathy into a congruent action response) (Carkhuff, 1969a, 1969b). The Carkhuff scales can require either a written or verbal response to a written statement or audio/video vignette, although instruction was originally mediated through audio recordings (Toukmanian & Rennie, 1975). Independent raters evaluate the level of empathy shown, selecting from five levels whereby level one represents low levels of empathy and level five indicates high levels. Level three is considered to be a minimally facilitative empathic response.

Using a slightly adapted version of the written statements format of the Carkhuff (1969b) scale, Larsen and Hepworth (1978) assessed students’ skill levels in providing empathic responses to ‘written messages’, which they suggest was highly significant (*p* < 0.001). We calculated a large effect size (1.51), demonstrating as predicted, that the experimental groups surpassed the control groups on achieved levels of performance.

Toseland and Spielberg (1982) sought to replicate and expand on Larsen and Hepworth's (1978) study by developing and evaluating a training programme comprising core helping skills, including genuineness, warmth and empathy. Two of the measures they used capture the initial skills outcome. First, through Carkhuff's Communication Index, as described above, students were asked to act as though they were the worker and respond by writing what they would say to a set of statements. Second, through part 1 of a Counselling Skills Evaluation (CSE), students watched a film of client vignettes, and wrote what they would say if they were the worker. Student responses to both measures were rated by trained judges. Students in the control group saw a slight reduction in their skills on both measures whereas the intervention group demonstrated gains on both measures with large effect sizes of 1.40 on the Carkhuff Communication Index and 1.20 on part 1 of the Counselling Skills Evaluation. Students in receipt of the training increased their ability to communicate effectively using the ten helping skills.

Nerdrum and Lundquist (1995) suggest that because Larsen and Hepworth (1978) and Toseland and Spielberg (1982) reported ratings for total communication index rather than empathy specifically, that lower empathy scores may have been concealed. Certainly, the instructors in the study reported by Nerdrum and colleagues (Nerdrum, 1997; Nerdrum & Høglend, 2003; Nerdrum & Lundquist, 1995), which narrowly missed the inclusion criteria for this review, found that empathy was the most difficult of the facilitative conditions for students to grasp. In addition, methods of training and methods of measurement have been confounded in earlier studies, potentially leading to over inflated treatment effects (Nerdrum & Høglend, 2003).

To evaluate an interviewing skills course, Laughlin (1978), also using the Carkhuff instrument, sought to test self‐instructional methods, in which one experimental condition relied on self‐reinforcement whilst the other experimental condition received external reinforcement and feedback from an instructor. Both experimental groups produced greater learning gains after training than either of the two control groups. Interestingly, there was no significant difference between the gain scores of the two experimental groups. Laughlin (1978, p. 65) suggests that ‘self‐managed behavior change can, under certain circumstances, prove to be as efficacious as externally controlled systems of behavior change’. However, students in the self‐reinforcement group rated their own empathic responses, whereas the supervisor rated the responses of students receiving the other experimental condition. As Laughlin (1978) acknowledged, ‘the self‐instruction group may be considered a product of inaccuracy in the self‐evaluation process’ (p. 68). Other studies have identified that students often over or underestimate their abilities (Kruger & Dunning, 1999). Based on their mean gain scores, we calculated a large effect size of 1.22 between the experimental condition who received external reinforcement and feedback and the control group who received no instruction.

Vinton and Harrington (1994) also appear interested in the role of the self in student learning, and they too used the Carkhuff scales to investigate this issue. At post‐test, a large effect size (0.88) was observed between the ‘videotape self and other’ group and the controls. At one month follow‐up, Vinton and Harrington (1994) found the majority of students in the intervention groups reached the level Carkhuff deemed to be facilitative.

To compare the effects of roleplay and using participants’ own problems for developing empathic communication skills through facilitative training, Wells (1976) used a variant of Carkhuff (1969a) communication test in which students were asked to respond empathically in writing to four tape‐recorded helpee statements before training and to a different set of four statements after training. Contrary to Wells’ assertion that no differential effect between role‐play and ‘own problems’ procedures was identified and the suggestion that active experimentation of students in both groups explains their modest outcome gains, we found a large effect size of 0.84 at post‐test. This finding should be interpreted cautiously given it is based on just five students per group.

Collins (1984) used two written skills measures—the Carkhuff stems, using written client statements as stimuli and a Skills Assessment Measure (SAM), which uses an audio‐video client stimulus. Both measures seek to capture outcomes that can be categorised as initial skills. The mean scores on the Carkhuff stems at post‐test were slightly higher for lab trained students than lecture trained students. Effect sizes were 0.60, 0.78 and 1.13 for empathy, warmth and genuineness respectively. However, Collins (1984) reports that statistical significance was only reached for empathy, which he suggests might be because lecture and lab training prepare students for training on the relatively straightforward measure of producing written statements as responses to short client vignettes. Warmth and genuineness might be easier to demonstrate than empathy hence lecture‐based students could manage them satisfactorily.

Similar, but slightly higher findings were demonstrated through the Skills Acquisition Measure (SAM), wherein students were asked to respond in writing to a series of vignettes. They were advised that their responses should be based on what they would say if they were conducting the interview. Student responses to the SAM were scored by trained raters using the Carkhuff scales. The post‐test scores of lab‐trained students compared favourably with the lecture‐trained students. Large effect sizes of 1.21, 1.37 and 1.77 were found empathy, warmth and genuineness respectively. Collins (1984) concluded that findings from the Carkhuff stems and the Skills Acquisition Measure provide evidence that lab‐based training is more effective for teaching interpersonal interviewing skills for social work students than lecture‐based training.

Carkhuff (1969a) suggested similarities between responses to the stimulus expressions in written form and verbal form and responses offered in an actual interview with a client. However, it should be noted that this alleged equivalency of measures has been questioned throughout the literature. VanCleave (2007) noted that making an advanced verbal empathic response is arguably more challenging than producing written statements. In her study, expert raters used the Carkhuff's Index for Communication scripts (CIC) to evaluate the videotaped responses of students to actors who verbally delivered excerpts based on the Carkhuff stems. Tapes contained vignette responses, rather than role‐played sessions in their entirety. With a large effect size of 1.79, students in the intervention group demonstrated more empathy than the students who did not receive the empathy response training.

In summary, multiple studies demonstrated an increase in social work students’ communication skills, including empathy, following training. The results for actual skill demonstration are modest yet promising.

###### Compilation

The compilation of skills is the term coined by Kraiger et al. (1993) to refer ‘to the grouping of skills into fluid behaviour’ (Carpenter, 2005, p. 12). Methods for measuring the compilation of skills include students’ self‐rating of competencies and observer ratings of students’ communication skills in simulated interviews (Carpenter, 2011). Wilt (2012) argued that simulation fosters more in‐depth learning than discussions, case studies, and role‐plays, due to the location of the student in the role of the worker and real‐time decision‐making that includes ethical considerations.

In the study by Collins (1984), analogue interviews, which consisted of a 10‐min role‐play of a student in the worker role with a student in the client role, showed modest gains, whereby 23% of students in the lab group improved by 0.5, to a level which Carkhuff and Berenson (1976) suggested was the sign of an effective intervention. This was significantly lower than the 52% who showed 0.5 improvement on the Skills Acquisition Measure. However, Collins (1984) suggests that direct comparisons of the findings is problematic given the delay (of approximately 3 weeks) in students completing the analogue measures, which reduced the time gap between pre‐and‐post‐training scores. Despite this, improvements shown in the analogue interviews were still significant. When comparing the two interventions—lab versus lecture, the lab‐trained students demonstrated more skill than the lecture‐trained group, as demonstrated by very large effect sizes of 1.74 for empathy, 1.80 for warmth and 1.88 for genuineness.

Hettinga (1978) sought to measure the impact of videotaped interview playback with instructional feedback on student social workers interviewing skills. A tailor‐made instrument was used to measure self‐perceived interviewing competence (SPIC). At post‐test, the mean score for the combined intervention groups was 62.60 whereas for the control groups the mean score was 57.47. This finding was supported by moderate to large effect sizes of 1.10 for Section 1 and 0.64 for Section 2, albeit with small sample sizes. The significantly higher scores for the intervention group suggest that students’ self‐perceived interviewing competence was positively impacted by videotaped interview playback with instructional feedback. Hettinga (1978) acknowledged the problem of using self‐reports as a measure of skill accomplishment. This is considered further in Section 6.3.

Both methods (self‐ratings and observer ratings) were used in the study conducted by Schinke et al. (1978). Through 10‐min videotaped role‐play simulations at pre‐ and post‐test, expert raters assessed a range of verbal and non‐verbal communication skills demonstrated by students. The largest effect sizes were for forward trunk lean (1.36) and open‐ended questions (1.01). After completing the videoed role‐plays, students rated their own interviewing skills according to an adapted version of the Counselor Effectiveness Scale developed by Ivey and Authier (1971). The intervention group's mean change score of 37.083 was significantly higher than the control group's mean change score of 13.182, producing an effect size of 0.93.

Ouellette et al. (2006) employed similar methods‐a 10‐min videotaped role‐play simulation and student self‐rating scale‐to evaluate the actual acquisition of interviewing skills between students taught in a traditional face to face class and students using a Web‐based instructional format with no face‐to‐face contact with the instructor. Rated according to a Basic Practice Interviewing Skills scale, very few statistically significant differences were found between the traditional class and the online class. Significant differences were identified for only 2 of 21 specific interviewing skills ratings, with an effect size of 0.73 for attentiveness and 0.93 for being relaxed. The findings indicate that for two of the interviewing skills measured, the online students were slightly more proficient than their peers in the traditional class. In a semester survey questionnaire, including a four‐item subscale measuring students’ perception of their acquisition of beginning interviewing skills, Ouellette et al. (2006) found few statistical differences between the groups apart from the classroom group responded more favourably in terms of their perception of learning a lot from the pedagogical activities used to teach interviewing skills. The interviewing skills of an online class versus those taught in a traditional face‐to‐face classroom setting were ‘approximately equal’ on completion of an interviewing skills course (Ouellette et al., 2006, p. 68).

In the study reported by Greeno et al. (2017) and Pecukonis et al. (2016), which investigated motivational interviewing, students’ empathic skills were observed and rated from low (score of 1) to high (score of 5) using the Motivational Interviewing Treatment Integrity (MITI) questionnaire. This measure, specific to the treatment modality of the intervention, provides a global empathy score, which aims to capture all of the efforts the student/practitioner makes to understand the client's perspective and convey this understanding to the client. Greeno et al. (2017) found improvements were evident for the intervention group, who received live supervision with simulated clients. At post‐test, the authors observed a small effect size of 0.24. The intervention group maintained gains at follow up, hence Greeno et al. (2017) conclude, ‘results from the study cautiously lend evidence that suggests live supervision as a promising practice for teaching MI to social work students’ (p. 803). These findings are particularly important given this is one of only two outcomes across all of the included studies to receive a low risk of bias rating.

Referring to the same study, Pecukonis et al.'s (2016) trained MITI coders produced summary scores deriving from the following behaviour counts. They found that the change scores between the start of the intervention and follow‐up were 1.39 for the live supervision group and −0.85 for the TAU group, providing support that Live Supervision was effective in teaching the early stages of MI skills. For empathy, at post‐test, a small effect size of 0.24 was observed. For the percentage of Motivational Interviewing adherent behaviours, an effect size of 0.34 was identified. Differences were less pronounced for MI specific skills. The authors observed that the intervention group displayed trends of attaining higher levels of proficiency on MI specific skills compared with the TAU group. An exception to this trend was observed at post‐test for percentage of complex reflections,—effect size −0.25, although they had lost this gain by follow‐up. Pecukonis et al. (2016) identify that statistical significance was seen only for the MI area of reflection to question ratio, acknowledging that the study may be underpowered.

Rawlings (2008) compared the performance of direct practice skills of students entering an undergraduate social work course with students exiting the same course. Students completed a 15‐min video‐taped interview with a standardised client. Students’ performance was evaluated by independent raters using an adapted version of a 14‐item instrument, developed by Chang and Scott (1999) to rate basic practice skills including beginning, exploring, contracting, case management skills, and the core conditions of genuineness, warmth, and empathy. Exiting students scored higher than entering students on each practice skill set, with a large effect size of 1.85 for the overall total score.

Studies measuring the compilation of skills demonstrated modest gains in students’ communicative abilities, including general social work interviewing skills and the demonstration of expressed empathy.

#### 5.4.5 Level 3: Behaviour and the implementation of learning into practice

Collins (1984) was the only study in this review to include a behavioural outcome. Scores from client interviews, which consisted of tape‐recorded interviews with clients at the start of their field practicums, were compared to scores from the analogue role‐play interviews at the end of the training to investigate the transfer of skills into practice. There was a drop for lab‐trained students from their analogue role‐play scores to their client interviews—from 2.72 to 2.22 (*T* = 7.59) for empathy, 2.79 to 2.35 (*T* = 6.82) for warmth and 2.63 to 2.28 (*T* = 6.65) for genuineness. These findings suggest students did not transfer their learning from the laboratory into practice, which Collins (1984) suggests was because of measurement anxiety, problems with the measures and the fundamental differences between lab and fieldwork settings.

#### 5.4.6 Level 4a: Changes in organisational practice

None of the included studies addressed this outcome.

#### 5.4.7 Level 4b: Benefits to users and carers

None of the included studies addressed this outcome.

## 6 DISCUSSION

### 6.1 Summary of main results

The purpose of this systematic review was to identify, summarise, evaluate and synthesise the current body of evidence to establish if CST programmes for social work students are effective. Fifteen studies were included in this review. Most of the studies included in this review are dated, methodological rigour was weak, quality was poor, and the risk of bias was moderate to high/serious or had to be rated as incomplete due to limitations in reporting. Extreme heterogeneity exists between the primary studies and the interventions they evaluated, precluding the meaningful synthesis of effect sizes through meta‐analysis. The findings of this review are therefore limited and must be interpreted with caution.

The anticipated outcome of a positive change in the modification of perceptions and attitudes of students (including cognitive and affective changes) following training was not born out in the data. This may in part be a result of how these outcomes are conceptualised and measured, with self‐reports being particularly problematic. Of the 15 included studies in this review, two studies, reported in one paper (Barber, 1988) (*N* = 82) identified a negative outcome for the acquisition of knowledge, whereby trained students placed less value on responsive and unresponsive interviewing behaviour and were less accurate in their ability to predict clients’ reactions than their untrained counterparts. However, there was no convincing evidence to suggest that the teaching and learning of communication skills in social work education causes adverse or harmful effects.

For the outcome of skills acquisition, which featured in 12 of the included studies, reported in thirteen papers, only one study (Ouellette et al., 2006) (*N* = 30), which compared face‐to‐face and online instruction, did not find a significant difference between the groups. Effect sizes in the other 11 studies measuring skills acquisition (Collins, 1984; Greeno et al., 2017; Hettinga, 1978; Larsen & Hepworth, 1978; Laughlin, 1978; Pecukonis et al., 2016; Rawlings, 2008; Schinke et al., 1978; Toseland & Spielberg, 1982; VanCleave, 2007; Vinton & Harrington, 1994; Wells, 1976) (*N* = 575) indicated some identifiable improvements in the communication skills including empathy, in students who received training. This finding is in keeping with reviews about CST (Aspegren, 1999) and empathy training (Batt‐Rawden et al., 2013) for medical students and nursing students (Brunero et al., 2010).

The review identified considerable gaps within the evidence, further research is required. This is discussed in Section 7.

#### 6.1.1 Level 1: Learner reactions

The evidence was inconclusive as only two studies (*N* = 108) contributed data. However, the findings, whilst limited, reflect a criticism of the growing trend, in the UK at least, to rely on quality assurance templates, which collect end of course satisfaction ratings only, and fail to measure outcomes (Carpenter, 2011).

#### 6.1.2 Level 2a: Modification in attitudes and perceptions

One study (*N* = 23), Schinke et al. (1978) found that students’ positive attitudes towards their skills were almost three times higher among students who had received CST than those who had not. Whilst promising, the evidence was inconclusive because too few studies contributed data. The review also highlights the challenges of using self‐reports to measure empathic understanding; no statistically significant changes were identified in three of four studies investigating empathic understanding, despite the same studies demonstrating the positive gains established when utilising other outcome measures. The challenges of measuring empathy through self‐reports (Lietz et al., 2011) are well documented and discussed further in Section 6.3.

#### 6.1.3 Level 2b: Modification in knowledge

The evidence was inconclusive, because only three studies (reported in two publications) (*N* = 150) contributed data. In a review of empathy training evaluation research, Lam et al., 2011 found that regardless of the training method used, individuals were able to learn about the concept of empathy. Whilst the modification of knowledge is relatively straightforward, this was evidently not an outcome reported in the studies in this review.

#### 6.1.4 Level 2b: Modification of skills

The evidence does suggest that modest gains can be made in the interviewing skills and the demonstration of empathic abilities of student social workers following systematic CST. This was the strongest finding of this review with 12 out of the 15 studies (*N* = 605) contributing data, 11 of which reported improvements for students in the intervention groups.

#### 6.1.5 Level 3: Changes in behaviour

The evidence was inconclusive due to the fact only one study (*N* = 67) reported this outcome.

#### 6.1.6 Level 4: Changes in organisational practice and benefits to users and carers

The outcomes was not addressed in any of the studies included in this review.

#### 6.1.7 Adverse effects

The evidence was inconclusive as only one paper (*N* = 82) contributed data.

### 6.2 Overall completeness and applicability of evidence

The included studies indicate, albeit tentatively, that interventions for teaching communication skills in social work education seem to have a positive impact, at least on demonstrable skills outcomes, and in the short‐term. Only Barber (1988) based on his own empirical research, questioned whether microskills were worth teaching. Perhaps the starkest finding of the review is the paucity of high quality and rigorously designed studies intended to present evidence for the outcomes of teaching communication skills to social work students, particularly given that pedagogic practices in the teaching and learning of communication skills are well established in social work education across the globe. Many of the included studies are quite dated and the majority were conducted in the United States. The picture provided by the existing body of evidence is incomplete‐it does not reflect the involvement of people with lived experience, or the newer innovations or technological advances used in social work education today‐limiting the applicability of the evidence.

In terms of publication bias, we recognise that there will be some PhD theses and trials containing negative results which we have not located in this review, and we acknowledge that publication bias could potentially be an issue. We took steps to minimise the risks including a wide reaching and extensive search (excluding outcomes) and contacting subject experts to identify any publications we might have missed through our search strategy. Strategies typically used to assess publication bias, such as funnel plots, were not feasible due to their small size and number, and lack of power.

Extreme levels of heterogeneity and moderate to high/serious risk of bias ratings in the studies included in the review, meant meta‐analysis was not feasible, and consequently a narrative review was undertaken. Outcomes were analysed and structured according to the outcomes framework for social work education developed by Carpenter (2005), after Kirkpatrick (1967), Kraiger et al. (1993) and Barr et al. (2000). Although data exists for some outcomes in levels 1–3, none of the included studies addressed outcomes at level 4a—changes in organisational practice or level 4b—benefits to users and carers, therefore significant gaps in the evidence base remain.

### 6.3 Quality of the evidence

Whilst there was overall consistency in the direction of mean change for the development of communication skills of social work students following training, we must acknowledge that the body of evidence is small in terms of eligible studies and that rigour across this body of evidence is low. The assessment of methodological quality and the risk of bias, examined using the ROB 2 tool for randomised trials and the ROBINS‐I tool for non‐randomised study, was judged to be moderate to high/serious, or incomplete, in all but one of the included studies. Confounders such as differences at baseline, missing data and the failure to address missingness appropriately, and the knowledge outcome assessors had about the intervention and its recipients were the most significant detractors from the internal validity of the studies reviewed.

Empathy has featured in skills training for more than 50 years, however as the studies in this review indicate, ‘evidence of empathy training in the social work curriculum, remains scarce and sketchy’ (Gerdes & Segal, 2011, p. 142). As Gair (2011, p. 791) maintains, ‘comprehensive discussion about how to specifically cultivate, teach and learn empathy is not common in the social work literature’, and the evidence that does exist is fairly limited. The same criticisms have been levied against research into the teaching and learning of communication skills in social work education more generally (Dinham, 2006; Trevithick et al., 2004). Given the range and extent of bias identified within this body of evidence, caution should be exercised in judging the efficacy of the interventions for improving the communicative abilities of social work students.

#### 6.3.1 Concerns about definitions and conceptualisations

One of the challenges evident in this review is the considerable variation in the way the study authors define key constructs, particularly in relation to empathy. Defining empathy remains problematic (Batt‐Rawden et al., 2013) because the construct of empathy lacks clarity and consensus (Gerdes et al., 2010) and conceptualisations have changed over time. Whilst cognitive, neurobiological, behavioural, and emotional components are now recognised (Lietz et al., 2011), earlier conceptualisations were more unidimensional, depicting empathy as a trait, emotion or skill. As a result, there is no consistency in the way operational definitions of empathy are used between the studies in this review, which has further implications for how outcomes are measured and restricts what the body of evidence can confidently tell us. The issue is not unique to social work; referring to a health context, Robieux et al., 2018, p. 59) suggest that ‘research faces a challenge to find a shared, adequate and scientific definition of empathy’.

#### 6.3.2 Concerns about measures

Communication skills, including empathy, can be measured from different perspectives including self‐rating (first person assessment), service user/patient‐rating (second person assessment) and observer rating (third person assessment) (Hemmerdinger et al., 2007). Ratings from service users were absent from the included studies, possibly because of geographical factors. Most of the included studies were conducted in North America where the inclusion of service users and carers in social work education is less prominent than in the UK, for example. Many of the included studies used validated scales whereas others developed their own measures. However, even with validated scales, measurement problems were encountered by the study authors.

##### Self‐rating

Much of the outcome data in social work education has relied on self‐report, a trend reflected in this review. Self‐reports appeared appropriate for measuring satisfaction with teaching and practice interventions in Laughlin (1978) and Ouellette et al.'s (2006) studies, although these outcomes did not correlate to student's improvement in skills. Self‐efficacy scales are another type of self‐report, one which has been adapted for research into the teaching and learning of communication skills of social work students specifically (e.g., Koprowska, 2010; Lefevre, 2010; Tompsett, Henderson, Gaskell Mew, et al., 2017b). They are inexpensive and easy to administer and analyse. However, the limitations of using self‐efficacy as an outcome measure are widely acknowledged (Drisko, 2014). Response‐shift bias is one limitation of self‐efficacy scales discussed in the literature, whereby some individuals may change their understanding of the concept being measured during the intervention. Such ‘contamination’ of self‐efficacy scores (Howard & Dailey, 1979) can mask the positive effects of the intervention. This may explain why no change was identified by Pecukonis et al. (2016); however since a retrospective pre‐test was not issued to the students in their study, neither the presence nor impact of response‐shift bias can be established. Alternatively, the scales themselves may have contributed to the surprising results found by Rawlings (2008) and Pecukonis et al. (2016) since neither were properly validated. The subjectivity of self‐efficacy scales has been identified as another area of concern. Previous research has found that students’ self‐ratings do not necessarily correlate with those of field instructors/practice educators (Fortune et al., 2005; Vitali, 2011), lecturers or service user‐actors (Koprowska, 2010). In this review, self‐efficacy scores and externally rated direct practice scores did not correlate in Rawlings (2008) study.

Self‐report instruments are still the most common way to measure empathy (Ilgunaite et al., 2017; Segal et al., 2017). However, the challenges associated with measuring perceived empathy through self‐reports (Lietz et al., 2011; Robieux et al., 2018) was clearly demonstrated in this review. Study authors anticipated that students’ perceived empathy levels would increase following training, but this expectation did not come to fruition in at least three studies, despite the study authors using different self‐report measures (including the IRI, QMEE and the TEQ), and even where other measures in the same studies did indicate skill gains. High and perhaps inflated ratings at pre‐test mask the improvements researchers anticipated. Greeno et al. (2017) acknowledged that training may impact more on behaviours and skills than self‐perception and identified that students’ TEQ scores were affected by high levels of perceived empathy at pre‐test. They suggested social desirability, meaning social work students want to be regarded as empathic, could compound this further, resulting in high rating scores at pre‐test. This ‘ceiling and testing effect’ (Greeno et al., 2017, p. 803) has been identified elsewhere (Gockel & Burton, 2014) and might result in a lack of significant changes in students’ level of reported empathy over time. Ilgunaite et al. (2017, p. 14) also warn of social desirability, highlighting the controversy associated with asking people with poor empathic skills to self‐evaluate their own empathic abilities.

Concerns have been raised about what self‐reports actually measure, reflecting one type of conceptualisation at the expense of others. For example, the Toronto Empathy Questionnaire used in Greeno et al.'s (2017) study views empathy primarily as an emotional process but leaves the cognitive components of perspective taking and self/other awareness unaccounted for. This reflects wider concerns regarding the validity of self‐report questionnaires as an accurate measure of outcomes.

The finding that self‐report scores did not significantly correlate with other measures that were used alongside them lends support to the claim that empathic attitudes are not ‘a proxy for actions’ (Lietz et al., 2011, p. 104). It is possible that skills training has more impact on students’ behaviours than their attitudes, a point that was made by Barber (1988). Regardless of the varying explanations, self‐report measures of empathy tell us very little about empathic accuracy (Gerdes et al., 2010, p. 2334). The problems are not specific to the studies in this review or social work education in general. In an evaluation of empathy measurement tools used in nursing research, Yu and Kirk (2009) suggested that of the 12 measures they reviewed, none of them were ‘psychometrically and conceptually satisfactory’ (p. 1790).

Schinke et al.'s (1978) study bucked the trend, finding students’ positive attitudes towards their skills were almost three times higher among those who had received CST compared to those who did not. Interestingly, the self‐report instrument used in this study measured clearly specified counselling skills, and thus did not suffer from the conceptual confusion faced by those seeking to measure empathy.

##### Observer ratings

Observer ratings, conducted by independent raters, are often considered to be more valid and reliable measures of communication skills than the aforementioned subjective self‐report measures. Observation measures enable third party assessment of non‐verbal and verbal behaviours to be undertaken. As Keefe (1979, p. 31) suggests, ‘accurate’ empathy when measured against a set of observer rating scales has been the basis for much valuable research and training in social work, particularly when combined with other variables. Observation measures were the primary instrument employed by the researchers of the included studies and produced the clearest demonstration of the effects of CST.

Studies using objective measures showed positive change, suggesting empathy training is effective. Studies using both self‐report and objective measures reported no significant changes in empathy using self‐report but found higher levels of behavioural empathy when using objective measures. The same pattern was identified in a review of empathy training by Teding van Berkhout and Malouff (2015). As Greeno et al. (2017, p. 804) explain, perceived empathy is not correlated to actual empathic behaviours *as scored by observers*. Observation measures also posed some challenges for the studies included in this review, for example the repeated use of scales in training and assessment creates the problem of test‐retest artefacts (Nerdrum & Lundquist, 1995).

The Carkhuff (1969a, 1969b) scales have been frequently used in social work education (Hepworth et al., 2010). The Carkhuff communication index is a written skills test measure used to assess the level of facilitative communication or core condition responses in relation to client statements of standardised vignettes. Carkhuff (1969a) reported that there is a close relation between responses to the stimulus expressions in written form and verbal form and responses offered in an actual interview with a client. Thus, Carkhuff concludes that ‘both written and verbal responses to helpee stimulus expressions are valid indexes of assessments of the counselor in the actual helping role’ (Carkhuff, 1969a, p. 108). However, mastery of accurate discrimination has not been sufficient to guarantee congruent empathic responding within a given verbal interaction. Providing verbal empathic responses is arguably more challenging than producing written statements, hence in VanCleave's (2007) study, trained raters used the Carkhuff's Index for Communication to score the empathic responses of students to the Carkhuff stems, which were delivered by trained actors. Through comparing the findings produced by different methods of measurement, Collins (1984) found, ‘students were significantly better at writing minimally facilitative skill responses than demonstrating them orally as measured in a role‐play interview (p. 124). Noting ‘a lack of equivalence between written and oral modes of responding’, the validity of the Carkhuff stems is challenged by Collins’ study (Collins, 1984, p. 148). Schinke et al. (1978) acknowledge similar concerns. Written skills test measures are not generalisable to, or indicative of, students’ behavioural responses in real life settings, threatening the ecological validity of such measures.

Vinton and Harrington (1994) also used the Carkhuff scale to measure expressed empathy and encountered measurement issues, which they suggest could have been caused by the validity of the measure, the additional statement they included in the questionnaire or other variables such as personality characteristics or background experiences.

The challenge of measuring empathy is apparent both within and across the included studies. Studies of empathy within social work have adopted a range of disparate methods to measure empathy depending on how it has been conceptualised (Lynch et al., 2019; Pedersen, 2009), often focusing on one component of empathy at the expense of another. As Gerdes and Segal (2009, p. 115) explain, ‘semantic fuzziness, conceptualizations and measurement techniques for empathy vary so much that it has been difficult to engage in meaningful comparisons or make significant conclusions about how we define empathy, measure it, and effectively cultivate it’.

#### 6.3.3 Concerns about outcomes

The paucity of evidence‐supported outcome measures in social work education has been apparent for some time (Holden et al., 2017), an issue we see reflected in this review.

##### Self‐efficacy

Self‐efficacy has been introduced as one means of assessing outcomes in social work education (Bell et al., 2005; Holden et al., 1997, 2002, 2005; Unrau & Grinnell, 2005). Self‐efficacy is deemed to be an important component of learning because ‘unless people believe they can produce desired effects by their actions, they have little incentive to act’ (Bandura, 1986, p. 3). However, the use of self‐efficacy as an outcome measure in social work education is not without controversy, with some people recommending that ‘change in actual behaviours should be assessed where possible’ (Doyle et al., 2011, p. 105). Rawlings (2008) cautions against the use of self‐efficacy as a proxy measure for skill; ‘measures of social work self‐efficacy are limited to student beliefs or perception regarding skill and do not measure actual performance’ (pp. 7–8).

#### 6.3.4 Concerns about research designs

The research designs used to investigate the effectiveness of interventions in social work education lack rigour, with few adhering to all the key features constituting a true experimental design. As Carpenter (2005, p. 4) suggests, ‘the poor quality of research design of many studies, together with the limited information provided in the published accounts are major problems in establishing an evidence base for social work education’ (Carpenter, 2005, p. 4). Identifying a dearth of writing which addressed the challenging issues of evaluating the learning and teaching of communication skills in social work education, Trevithick et al. (2004, p. 28), in a UK‐based review, point out that ‘without robust evaluative strategies and studies the risks of fragmented and context restricted learning are heightened’. Similar issues arise in educational research more generally.

#### 6.3.5 Concerns about researcher allegiance, positionality and confirmation bias

The study authors are predominantly social work academics conducting research within their own institutions. It is highly likely that they will have a vested interest in wanting the teaching of communication skills to be successful, particularly if they have been involved in the development of the intervention(s) under investigation. Researcher allegiance bias, and the challenges this presents are increasingly being recognised (Grundy et al., 2020; Montgomery & Belle Weisman, 2021; Uttley & Montgomery, 2017; Yoder et al., 2019). Whilst some risks of bias have been reduced within the included studies, they have not been eliminated. The relationships between students, academics and researchers, and the impact these dynamics may have on study findings, are largely under‐explored.

The studies included in this review are not large multi‐team trials, rather the study authors are working in small groups or alone, which hampers the resources available to them to mitigate bias in data collection and analysis procedures. Using an independent statistician to facilitate the blinding of outcome measures would have enabled study authors to overcome the inability to blind the participants or the experimenters.

Reviewers are no more immune from conflicts of interest or unconscious bias than the triallists and researchers of the included studies. Both reviewers are social work academics, and the first author (ERH) teaches communication skills to social work students, which is why it became the topic of her PhD. Whilst neither reviewer have a vested interest in any of the authors, institutions or interventions under investigation in the included studies, the first author acknowledges that she believes, or at least hopes, that students’ communication skills and their development of empathy, will be enhanced through taught interventions. ERH has had to be very mindful throughout the review of the potential for unconscious confirmation bias, and the need to remain as objective and impartial as possible. She also recognises that her own positionality, influenced by pedagogic experiences and social work values, have led her to believe in the importance of the educator's teaching style, the positive contribution of service user and carer involvement, and the added value of involving students in curriculum delivery and design, especially for developing social work skills (Reith‐Hall, 2020). These components were largely absent from the included studies, a source of frustration to the first author, who frequently has to remind herself that constructs of teaching and learning have changed considerably from when the majority of the included studies were undertaken, and that her views on such matters might be partly cultural and highly personal. Whilst unlikely to have affected the conduct or findings of the review itself, ERH recognises her beliefs have a bearing on the gaps identified in the research and potential policy and practice implications.

### 6.4 Potential biases in the review process

We performed a comprehensive search of a wide range of electronic databases and grey literature followed by the hand searching of key journals and reference searching of relevant studies. Both members of the review team screened all records and assessed all included studies against the inclusion criteria set out in the protocol, increasing consistency and rigour and minimising potential biases in the review process.

We sought to locate all publicly available studies on the effect of teaching and learning of communication skills in social work education during the review process, however it is difficult to establish if our endeavours were successful. It was a surprise to the first author that one of the included studies, which very clearly met the inclusion criteria, was obtained through reference searching, rather than through the electronic database search. As predicted by the second author, the age and style of the publication meant no key words were used, a search function upon which the electronic databases rely. Whilst this study came to light through reference searching, we cannot be entirely sure that other similar studies were surfaced in this way. Therefore publication bias cannot be entirely ruled out.

Our search was not limited to records written in English; indeed, one of the two unobtainable studies was written in Afrikaans, however, the rest of the studies were written in English. Rather than indicating a limitation of the way the review was conducted, it is likely that the location of the studies is responsible for the language bias—all of the included studies were conducted in English‐speaking countries, with the majority from the United States. Evidence‐based practice is well established in the United States, contributing to the use of study designs that increase the likelihood of them being included in systematic reviews.

Uncertainties and differences of opinion were resolved through contacting study authors for further information and through further reading and discussion, without recourse for a third‐party adjudicator. Both reviewers independently screened and assessed the studies. We are not aware of other potential biases or limitations inherent within the review process.

### 6.5 Agreements and disagreements with other studies or reviews

Findings from the included studies indicate that communication skills including empathy can be learned, and that the systematic training of student social workers produces improvements in their communication skills (Greeno et al., 2017; Larsen & Hepworth, 1978; Laughlin, 1978; Pecukonis et al., 2016; Schinke et al., 1978; VanCleave, 2007), at least in the short term.

The findings of this systematic review broadly agree with the knowledge reviews about communication skills produced for the Social Care Institute of Excellence (Luckock et al., 2006; Trevithick et al., 2004). The knowledge reviews highlight that despite a lack of evidence, weak study designs, and a low level of rigour, study findings for the teaching and learning of communication skills in social work education are promising. Reviews of communication skills and empathy training in medical education (Aspegren, 1999; Batt‐Rawden et al., 2013), where RCTs and validated outcome measures prevail, also suggest that CST leads to demonstrable improvements for students.

The findings from our review identified the same gaps as those found in the UK‐based social work knowledge and practice reviews for social work education, suggesting that little has changed. Trevithick et al. (2004) suggest that interventions are under‐theorised and the issue of whether students transfer their skills from the classroom to the workplace is unclear. Our findings concur with these observations. Diggins (2004) and Dinham (2006) identified the existence of far greater expertise and more examples of good practice than that reflected in the literature. Regrettably, our review suggests little has changed in almost 20 years.

## 7 AUTHORS’ CONCLUSIONS

### 7.1 Implications for practice

This review aimed to examine effects on a range of outcomes in social work education. With the exception of skill acquisition, there was insufficient evidence available to offer firm conclusions on other outcomes. It is unclear whether an issue with measurement or something to do with how students learn, or a combination of the two, is responsible for such uncertainty. Our understanding of how communication skills and empathy are learnt and taught remain limited, due to a lack of empirical research and comprehensive discussion. Discussing pedagogical explorations of empathy, Zaleski (2016, p. 48) points out, ‘there lacks a sufficient exploration of specific teaching strategies’. Our review echoes and amplifies this view, within the context of social work education specifically. Disagreement remains within social work academia as to what empathy consists of. Segal et al. (2017) draw on cognitive neuroscience, and the role of mirror neurones, to underpin the teaching of empathy in social work education and practice. Eriksson and Englander (2017, p. 607) take ‘a critical, phenomenological stance towards Gerdes and Segal's work’, exploring how empathy is conveyed in a context where practitioners are unlikely to be able to relate personally to the experiences of their client group. Given the continuing debate about the role of walking in someone else's shoes, it is hardly surprising that the studies in this review conceptualise and measure different aspects of empathy in a variety of ways producing incomplete and inconsistent results. Due to the clinical heterogeneity of populations and interventions, low methodological rigour and high risk of bias within the included studies, caution should be exercised when interpreting the findings for practice and policy.

Despite the limitations and variations in educational culture, the findings are still useful, and indicate that CST is likely to be beneficial. One important implication for practice appears to be that the teaching and learning of communication skills in social work education should provide opportunities for students to practice skills in a simulated (or real) environment. Toseland and Spielberg (1982) suggest that skills diminish gradually if not reinforced. They suggest that students should be exposed to the effective application of interpersonal helping skills in several different courses and be encouraged to practice these skills in a variety of case situations role‐played in classroom and laboratory settings, as well as in field settings. Larsen and Hepworth (1978) and Pecukonis et al. (2016) also suggest that CST must be better integrated with practice settings, where students can demonstrate communicative and interviewing abilities with actual clients in real‐world practice settings, ‘the ultimate test of any social work practice skill’ (Schinke et al., 1978, p. 400).

Technology is widely used in the teaching and learning of communication skills in social work education, and whilst technological advances have been considerable in recent years, current practice is not captured in the studies featuring in this review. The further sharing of good practice between students and educators continues to be necessary. The Australian Association of Social Workers identifies that face‐to‐face teaching remains the standard approach for teaching professional practice skills, whilst acknowledging that online technologies and blended learning are also encouraged (Australian Association of Social Workers, 2020). Barriers preventing the further uptake of technology throughout social work education have been identified. In a review of the literature into key issues with web‐based learning in human services, Moore (2005) discovered that some social work educators believe traditional instruction to be superior to web‐based instruction, especially for courses focused on micro practice and clinical skills. Similar findings have been reproduced more recently, especially for practice‐oriented competencies (Levin et al., 2018). Despite such reservations, reviews into technology‐based pedagogical methods in social work education have indicated that students’ competencies were largely equitable between online and face‐to‐face modalities (Afrouz & Crisp, 2021; Wretman & Macy, 2016). The extent to which this applies to outcomes of communication skills and empathy remains unknown. In this review the studies that compared face‐to‐face interventions with online interventions did not reach a consensus, since Ouellette et al. (2006) found there was no difference in outcomes between online and face‐to‐face teaching, whilst Greeno et al. (2017) and Pecukonis et al. (2016) found the outcomes of students who received live supervision were greater than those who engaged in self‐directed study online. However, we do not know whether student outcomes were affected by the presence or absence of an educator. Differences might not be attributable to the interventions themselves, for as Levin et al. (2018, p. 777) remark, ‘the role of an instructor in online learning cannot be underestimated’.

Certainly, the proliferation of online social work courses is evident across Australia (Australian Association of Social Workers, 2018) and the USA (Council on Social Work Education, 2020). The global coronavirus/Covid‐19 pandemic has led to exponential growth of online teaching and learning in social work education, hence ‘we can be nearly certain that the ‘new normal’ will include the use of information technology’ (Tedam & Tedam, 2020, p. 3). Therefore, it is imperative that we investigate the impact of online learning and web‐based instruction and the role of the educator in different contexts on the development of social work students’ communicative and empathic abilities.

### 7.2 Implications for research

There is much to be done to improve the outcome studies in social work education generally and for the teaching and learning of communication skills in social work education specifically. Robust study designs that support causal inferences through the random allocation to intervention and control groups is a necessity. Steps to reduce threats to the internal validity of case‐controlled studies should also be exercised to reduce the impact of test–retest artefacts identified by Nerdrum and Lundquist (1995) in some of the other studies. More work is needed on defining and measuring outcomes (Diggins, 2004). Validated measures which can be used consistently across future studies would make comparisons easier and enable future synthesis to be more meaningful.

The review found that relying solely on self‐report measures was problematic, particularly given that the findings from these did not correlate with the findings produced from other measures. Vinton and Harrington (1994) found there was no statistically significant correlation between students’ perceptions of their learning experience and self‐assessment of their skill acquisition with the independent evaluator's rating of the students’ acquisition of interviewing skills. Methodological triangulation should be considered in future studies.

Other study authors advise researchers to use objective measures of communication skills including behavioural measures of empathy (Greeno et al., 2017; Pecukonis et al., 2016), a recommendation also made by Teding van Berkhout and Malouff (2015) in a review of empathy training. Collins (1984) recommended that more research is required on the equivalency of measures, given the different results the measures in his study produced. Carpenter (2005, 2011) provides guidance on how research designs and outcome measures can be further developed in social work education. This review highlights the need for research that utilises follow‐up studies, which would help determine the extent to which training benefits endure after the end of training (Schinke et al., 1978; VanCleave, 2007). Rawlings (2008) advises that a longitudinal design, testing the same students over time, is required. The need to investigate whether or not students were able to transfer their skills into practice has also been firmly stated (Carpenter, 2005).

In addition to outcome studies, VanCleave (2007) recommends the inclusion of qualitative data in researching the teaching and learning of communication skills in social work education. Building a qualitative strand into the research design would facilitate exploration and explanation of the quantitative outcomes. It would also enable the voices of the intended beneficiaries of the interventions under investigation to be heard and acted upon. As a values‐based profession, a focus on stakeholder participation and contribution should be at the forefront of research in social work education. The benefits of involving service users and carers in social work education are well rehearsed, and examples of their input in the teaching and learning of communication skills are plentiful within the wider literature. However, the value of service users and carers is not evident within the included studies, thus gap‐mending strategies need to be established across the realms of social work education, practice and research, to prevent certain types of social work knowledge receiving more preferential status than others. As Carpenter (2005, p. 7) points out, since the purpose of the whole exercise is to benefit service users and/or carers, a comprehensive evaluation should ask whether training has made any difference to their lives’.

Finally, the theory of change appears to be assumed rather than clearly defined. Research that identifies the relevant substantive theories on which the teaching and learning of communication skills is based would provide a good starting point. Moreover, whilst the studies in the review indicated that CST encourages some improvement, particularly in terms of the skills outcomes measured, clarity on the mechanisms involved in positive effects requires additional research. The role of reflection, whilst briefly mentioned in some of the included studies, has been largely overlooked. The role of context is almost completely absent in the existing body of literature. Zaleski (2016) suggest the teaching style of the educator can influence students’ ability to learn empathy, yet they acknowledge that literature into the educational environment is lacking. A realist synthesis would support the theoretical development of the teaching and learning of communication skills in social work education. Realist synthesis is an interpretive theory‐driven methodological approach to reviewing quantitative, qualitative and mixed methods research evidence about complex social interventions to provide an explanatory analysis of how and why they work in particular contexts or settings. This research approach would support the theoretical development of the teaching and learning of communication skills in social work education, complementing that of this systematic review (Reith‐Hall, 2022).

## CONTRIBUTIONS OF AUTHORS

- Content: Emma Reith‐Hall
- Systematic review methods: Emma Reith‐Hall and Paul Montgomery
- Statistical analysis: Paul Montgomery
- Information retrieval: Emma Reith‐Hall
- Write up: Emma Reith‐Hall and Paul Montgomery

## DECLARATIONS OF INTEREST

Emma Reith‐Hall is a social work academic who has been involved in the teaching and learning of communication skills in social work education in a number of higher education institutions. The author acknowledges she holds a position whereby she believes that communication skills can, and should, be taught, learnt, and refined. Paul Montgomery is primarily a methodologist and systematic reviewer who considers his position on the issue of communication skills to be equivocal. Neither author has a financial conflict of interest.

## DIFFERENCES BETWEEN PROTOCOL AND REVIEW

None.

## SOURCES OF SUPPORT

**Internal sources**

- Emma Reith‐Hall and Paul Montgomery, UK
  No internal sources of support.
**External sources**
- Emma Reith‐Hall, UK
  ERH is undertaking the systematic review as part of her PhD research, for which she receives ESRC DTP funding (Grant number: ES/P000711/1).
- Paul Montgomery, UK

No sources of support

## Supporting information

Supporting information.
