# Supplementary material for: Communication skills training for improving the communicative abilities of student social workers
Source: Campbell Syst Rev. 2023 Feb 23;19(1):e1309. doi: 10.1002/cl2.1309 (PMC9949884; doi:10.1002/cl2.1309)
Supplement: Supplementary file 1 — Supporting information. [file CL2-19-e1309-s001.docx]

Appendices

1 Example Search Strategy

“social work student” OR “social work students” OR “student social worker” OR “student social workers”

AND

Train* OR educat* OR teach* OR course OR module OR School* OR Learn*

AND

Communic* OR Interpersonal OR Interview* OR microskills

**Example of the search used for Medline**

| 1. exp social work student/ |
| --- |
| 2. student social work*.mp. [mp=ti, ab] |
| 3. social work student*.mp. [mp=ti, ab] |
| 4. 1 or 2 or 3 |
| 5. exp communication skill/ |
| 6. communicat*.mp. [mp=ti, ab] |
| 7. exp interpersonal communication/ |
| 8. interpersonal.mp. [mp=ti, ab] |
| 9. exp interview/ |
| 10. interview*.mp. [mp=ti, ab] |
| 11. 5 or 6 or 7 or 8 or 9 or 10 |
| 12. exp training/ |
| 13. train*.mp. [mp=ti, ab] |
| 14. exp education/ |
| 15. educat*.mp. [mp=ti, ab] |
| 16. exp teaching/ |
| 17. teach*.mp. [mp=ti, ab] |
| 18. exp learning/ |
| 19. learn*.mp. [mp=ti, ab] |
| 20. exp curriculum/ |
| 21. curricul*.mp. [mp=ti, ab] |
| 22. 12 or 13 or 14 or 15 or 16 or 17 or 18 or 19 or 20 or 21 |
| 23. 4 and 11 and 22 |

## 2 Risk of Bias Tables

**Risk of Bias Tables for randomised studies**

**Risk of Bias Table for Hettinga (1978)**

| **Bias Domain** | **Bias judgement** | **Support for judgement** |
| --- | --- | --- |
| **Domain 1**  Risk of bias arising from the randomization process | Low risk | **Quotes:**  Students were instructed to randomly select a numbered folded paper from a brown paper bag (Hettinga, 1978, p. 39).  For the self-esteem outcome: the results of that analysis indicate little difference between treatment means on the self-esteem at the pre-test administration (Hettinga, 1978, p. 64).  For the self-efficacy outcome: no pre-tests were undertaken.  The equivalence of the two treatment groups, within each section prior to the actual period of treatment is assumed (Hettinga, 1978, p. 64).  **Comment:**  Hettinga used an odd/even system but then alternated which section received the intervention so we do not think the allocation sequence could have been predicted (see Hettinga, 1978, p. 39). |
| **Domain 2**  Risk of bias due to deviations from the intended interventions | Not reported | **Comment:**  There is no information about deviations from the intended intervention. |
| **Domain 3**  Risk of bias due to missing outcome data | High risk | **Quote:**  Over the course of the research project (which includes all four testings), 6 subjects were lost. From section 1, 3 experimental and 1 control subject provided incomplete data. From section 2, 1 experimental and 1 control subject provided incomplete data. The researcher assumes that no interaction of selection and mortality occurred and that consequently, the research results were not skewed (Hettinga, 1978, 57).  **Comment:**  The researcher’s assumption does not constitute evidence that missing outcome data does not pose a risk of bias.  **Comment:**  Missingness in the outcome could depend on its true value; no information is provided from which the likelihood of this issue arising can be judged. |
| **Domain 4**  Risk of bias in measurement of the outcome | High risk  (Self-perceived competence) | **Comment:**  There is no evidence to suggest that the method of measuring either outcome (self-esteem or self-efficacy) was inappropriate.  **Quote:**  The researcher had no knowledge as to what treatment group the subject was randomly assigned.  **Comment:**  Participants, by virtue of completing self-esteem and self-efficacy questionnaires, were the outcome assessors completing subjective measures. It is probable that students were aware of the intervention group to which they belonged. The outcomes could have been influenced by knowledge of the intervention, if for example, the self-instruction group were motivated to try harder to compete with the group led by an instructor. There was no information on which to judge the likelihood of this happening, but it could not be ruled out. |
|  | Some concerns  (Self-esteem) | **Quote:**  ‘It seems clear that on the average, the treatments studied did not affect the self-esteem scores of the subjects’ (p. 60).  **Comment:**  Given there was no differences in self-esteem ratings between the groups, we can be fairly confident that participants’ reporting of the outcome was not influenced by knowledge of the intervention they received. |
| **Domain 5**  Risk of bias in selection of the reported result | Not reported | **Comment:**  There is no evidence of a pre-specified analysis plan or protocol from which to judge whether bias arose in the selection of the reported results. |
| **Overall risk of bias** | High risk | **Comment:**  At least one domain was assessed as high risk, which means the overall bias rating was also high risk. |

**Risk of Bias Table for Larsen & Hepworth (1978)**

| **Bias Domain** | **Bias judgement** | **Support for judgement** |
| --- | --- | --- |
| **Domain 1**  Risk of bias arising from the randomization process | Low risk | **Quote:**  ‘The use of randomization controlled for group variability prior to the induction of the experimental condition’ (Larsen & Hepworth, 1978, p. 76). This implies allocation concealment, although it is not explicit.  **Quote:**  ‘Comparisons of the pre-experimental means further ascertained group equivalency, since the means of the four groups pretested were virtually identical’ (Larsen & Hepworth, 1978, p. 76).  **Comment:**  No concerns regarding baseline differences between groups. |
| **Domain 2**  Risk of bias due to deviations from the intended interventions | Not reported | **Comment:**  There is no information about deviations from the intended intervention. |
| **Domain 3**  Risk of bias due to missing outcome data | Low risk | **Comment:**  There was no attrition or missing data. |
| **Domain 4**  Risk of bias in measurement of the outcome | Low risk | **Comment:**  The study (Larsen and Hepworth, 1978) does not say if the outcome assessors knew which group the students belonged to. However, in Larsen’s (1975) PhD thesis, on which the published article is based, she says that three raters (social work practitioners) blindly rated a third of students each, ‘with no cues as to the identification of the student or the group to which he belonged’ (Larsen, 1975, 64). |
| **Domain 5**  Risk of bias in selection of the reported result | Not reported | **Comment:**  There is no evidence of a pre-specified analysis plan or protocol from which to judge whether bias arose in the selection of the reported results.  **Comment:**  Pre-tests for one control group are absent but this was part of the research design (to avoid the Hawthorne effect) (Larsen and Hepworth, 1978, p. 76). |
| **Overall risk of bias** | Low risk | **Comment:**  The overall bias rating was low risk |

**Risk of Bias Table for Laughlin (1978)**

| **Bias Domain** | **Bias judgement** | **Support for judgement** |
| --- | --- | --- |
| **Domain 1**  Risk of bias arising from the randomization process | Low risk | **Quote:**  ‘All subjects were randomly assigned using a table of random numbers’ (Laughlin, 1978, p. 40).  **Quote:**  At the end of the class session ‘the investigator handed out manila envelopes containing instructions and training materials’ (Laughlin, 1978, p. 48).  **Quote:**  Age, prior job and training experience were equivalent for all groups at the outset (Laughlin, 1978, p. 65). |
| **Domain 2**  Risk of bias due to deviations from the intended interventions | Not reported | **Comment:**  There is no information about deviations from the intended intervention. |
| **Domain 3**  Risk of bias due to missing outcome data | High risk | **Quote:**  Seven subjects (of 68) failed to complete either the pre- or post-test because of absence from class on the day these tests were administered.  **Comment:**  There was no evidence that the result was not biased by missing outcome data.  **Comment:**  Missingness in the outcome could depend on its true value, if students who perceived their communication skills as weak did not attend at post-test; no information is provided from which the likelihood of this issue arising can be judged. |
| **Domain 4**  Risk of bias in measurement of the outcome | High risk | **Comment:**  Students in the self-reinforcement group rated their own empathic responses, whereas the supervisor rated the responses of students receiving the other experimental condition.  **Quote:**  ‘Since the supervisor rated practice scores for Experimental Group I and subjects rated their own scores in Experimental Group II, the higher mean scores for the self-instruction group may be considered a product of inaccuracy in the self-evaluation process’ (Laughlin, 1978, 68). |
| **Domain 5**  Risk of bias in selection of the reported result | Not reported | **Comment:**  There is no evidence of a pre-specified analysis plan or protocol from which to judge whether bias arose in the selection of the reported results. |
| **Overall risk of bias** | High risk | **Comment:**  At least one domain was assessed as high risk, which means the overall bias rating was also high risk. |

**Risk of Bias Table for Greeno et al. (2017)**

| **Bias Domain** | **Bias judgement** | **Support for judgement** |
| --- | --- | --- |
| **Domain 1**  Risk of bias arising from the randomization process | Low risk | **Quote:**  ‘Then, using simple random assignment, they were randomized to treatment condition and received either training in LS (n = 26) or TAU (n = 28)’ (Greeno et al., 2017, p. 798).  **Comment:**  Email contact (received 26.04.2021) with lead study author (Elizabeth Greeno) confirmed that randomisation occurred using SPSS.  **Quote:**  There were no significant demographic differences between groups (Greeno et al., 2017, p. 798). |
| **Domain 2**  Risk of bias due to deviations from the intended interventions | Not reported | **Comment:**  There is no information about deviations from the intended intervention. |
| **Domain 3**  Risk of bias due to missing outcome data | Low risk | **Comment:**  Data were available for most participants. |
| **Domain 4**  Risk of bias in measurement of the outcome | Low risk  (Behaviour change) | **Quote:**  ‘Audiotapes only were coded by graduate research assistants (GRAs) to maximize the anonymity of the participants. Coders were blind to the participant’s condition and assessment time point’ (Greeno et al., 2017, p. 799).  **Comment:**  Outcome assessors were not aware of the interventions received by study participants. |
|  | Some concerns  (Perceived empathy) | **Quote:**  ‘There was not a statistically significant difference or change in self-reported empathy between the groups across time (p. 800).  **Comment:**  Given there was no difference in perceived empathy scores between the groups, we can be fairly confident that participants’ reporting of the outcome was not influenced by knowledge of intervention received. |
| **Domain 5**  Risk of bias in selection of the reported result | Not reported | **Comment:**  There is no evidence of a pre-specified analysis plan or protocol from which to judge whether bias arose in the selection of the reported results. |
| **Overall risk of bias** | Low risk  (Behaviour change) | **Comment:**  Low risk bias ratings were given for the domains where sufficient information was provided for a bias judgement to be made, hence the overall bias rating was also low risk. |
|  | Some concerns  (Perceived empathy) | **Comment:**  Because one outcome measure was assessed as having ‘some concerns’, the overall bias rating for this outcome measure is also ‘some concerns’. |

**Risk of Bias Table for Pecukonis et al. (2016)**

| **Bias Domain** | **Bias judgement** | **Support for judgement** |
| --- | --- | --- |
| **Domain 1**  Risk of bias arising from the randomization process | Low risk | **Quote:**  The students were then randomly assigned to either the LS (n = 26) condition or the TAU (n = 28) (Pecukonis et al., 2016, p. 486).  **Comment:**  Email contact (received 26.04.2021) with one of the study authors (Elizabeth Greeno) confirmed that randomisation occurred using SPSS.  **Quote:**  No statistical difference existed between groups on baseline proficiency levels (Pecukonis et al., 2016, p. 479). |
| **Domain 2**  Risk of bias due to deviations from the intended interventions | Not reported | **Comment:**  There is no information about deviations from the intended intervention. |
| **Domain 3**  Risk of bias due to missing outcome data | Low risk | **Comment:**  Data were available for most participants. |
| **Domain 4**  Risk of bias in measurement of the outcome | Low risk  (Behaviour change) | **Quote:**  Coders did not have any contact with study participants, and study data were masked to prevent coders from discerning students’ identity, group assignment, and when the interview occurred in the T2–T4 assessment process (Pecukonis et al., 2016, p. 486).  **Comment:**  Outcome assessors were not aware of the interventions received by study participants. |
|  | Some concerns  (self-efficacy) | **Quote:**  ‘For the self-efficacy survey, no statistically significant difference was found across time or between groups’ (p. 492).  **Comment:**  Given there was no difference in self-efficacy scores between the groups, we can be fairly confident that participants’ reporting of the outcome was not influenced by knowledge of intervention received. |
| **Domain 5**  Risk of bias in selection of the reported result | Not reported | **Comment:**  There is no evidence of a pre-specified analysis plan or protocol from which to judge whether bias arose in the selection of the reported results. |
| **Overall risk of bias** | Low risk  (Behaviour change) | **Comment:**  Low risk bias ratings were given for the domains where sufficient information was provided for a bias judgement to be made, hence the overall bias rating was also low risk. |
|  | Some concerns  (self-efficacy) | **Comment:**  Because one outcome measure was assessed as having ‘some concerns’, the overall bias rating for this outcome measure is also ‘some concerns’. |

**Risk of Bias Table for Schinke et al. (1978)**

| **Bias Domain** | **Bias judgement** | **Support for judgement** |
| --- | --- | --- |
| **Domain 1**  Risk of bias arising from the randomization process | Some concerns | **Comment:**  23 graduate student subjects were matched on sex and time spent in field practice, then assigned randomly to the intervention or control condition. There is no information about the method of randomization or allocation concealment. |
| **Domain 2**  Risk of bias due to deviations from the intended interventions | Not reported | **Comment:**  There is no information about deviations from the intended intervention. |
| **Domain 3**  Risk of bias due to missing outcome data | Low risk | **Comment:**  There was no attrition or missing data. |
| **Domain 4**  Risk of bias in measurement of the outcome | Low risk  (Behaviour change) | **Comment:**  Outcome assessors were not aware of the interventions received by study participants.  **Quote:**  ‘All videotaped interviews were scored by six trained raters, ignorant of study hypothesis and subject conditions’ (Schinke et al., 1978, 396). |
|  | Some concerns  (Perceived performance) | **Comment:**  The Counselor Effectiveness Scales was used by students to quantify their attitudes towards their own interviewing behaviour. It is likely that the delayed waiting list procedure reduced the impact of knowledge of intervention on the students. |
| **Domain 5**  Risk of bias in selection of the reported result | Not reported | **Comment:**  There is no evidence of a pre-specified analysis plan or protocol from which to judge whether bias arose in the selection of the reported results. |
| **Overall risk of bias** | Some concerns | **Comment:**  At least one domain was assessed as having ‘some concerns’, which means the overall bias rating was also ‘some concerns’. |

**Risk of Bias Table for Wells (1976)**

| **Bias Domain** | **Bias judgement** | **Support for judgement** |
| --- | --- | --- |
| **Domain 1**  Risk of bias arising from the randomization process | Some concerns | **Comment:**  The training procedures – role-play or own-problems were randomly assigned to groups - does not specify how randomisation occurred.  **Comment:**  No evidence to suggest baseline differences occurred between groups. |
| **Domain 2**  Risk of bias due to deviations from the intended interventions | High risk | **Comment:**  Two students swapped groups, the data for whom was analysed in terms of the interventions they received rather than the interventions they were initially assigned to. |
| **Domain 3**  Risk of bias due to missing outcome data | High risk | **Comment:**  4 out of 14 students did not complete post-test. There was no information about the reasons for this, although absences were equal between the groups.  **Comment:**  There was no evidence that the result was not biased by missing outcome data.  **Comment:**  Missingness in the outcome could depend on its true value, if students who perceived their communication skills as weak did not attend at post-test; no information is provided from which the likelihood of this issue arising can be judged. |
| **Domain 4**  Risk of bias in measurement of the outcome | Low risk | **Comment:**  Rater was blind to pre-test and post-test status and also to intervention group status therefore outcome assessor was not aware of the interventions received by study participants. |
| **Domain 5**  Risk of bias in selection of the reported result | Not reported | **Comment:**  There is no evidence of a pre-specified analysis plan or protocol from which to judge whether bias arose in the selection of the reported results. |
| **Overall risk of bias** | High risk | **Comment:**  At least one domain was assessed as high risk, which means the overall bias rating was also high risk. |

**Risk of bias tables for non-randomised studies**

**Risk of Bias Table for Barber (1988) Experiment 1**

| **Bias Domain** | **Bias judgement** | **Support for judgement** |
| --- | --- | --- |
| **Domain 1**  Bias due to confounding | Serious | **Comment:**  Barber acknowledges confounding based on the use of two different actresses, alongside suggested resolutions (see quote below).  **Quote:**  ‘One might object that since two different people displayed the two kinds of non-verbal behavior, nonverbal non-responsiveness had been confounded with some other qualities of the interviewers which account for the differences observed in expertness, trustworthiness, and attractiveness. However, the counselors were closely matched on all possible variables except for nonverbal responsiveness and other salient differences are difficult to imagine. In addition, steps were taken to control other variables. No sound was recorded; interviewers were videotaped from the same perspective, mouthing the same utterances’ (p. 8).  **Comment:**  There is no information about how the 16 first year and 16 final year students were recruited.  Barber acknowledges differences between groups but no measures to control for this were undertaken.  **Quote:**  First, the experimental procedure precluded random assignment to trained and untrained conditions. Instead, a group of already trained students was compared with a group of new arrivals at the university. It is quite probable, therefore, that the two groups differed in ways other than level of casework training. We cannot conclude unequivocally that microskills training alone is responsible for the differences reported. It may be due to a range of influences associated with the transition from first year to fourth year. Results may even have been influenced by differences in the kinds of individuals recruited to the two groups. Such explanations can never be ruled out when the experimental procedure entails non-random assignment to one or more conditions’ (p. 8)  **Comment:**  Attempts to control for confounding were insufficient, producing a serious risk of bias rating for experiment 1. |
| **Domain 2**  Bias in selection of participants into the studies | Low | **Comment:**  The information about sampling does not suggest that students were selected on the basis of participant characteristics observed after the start of the intervention. |
| **Domain 3**  Bias in classification of interventions | Low | **Comment:**  Bias in the classification of interventions is unlikely when the intervention occurs at population level. |
| **Domain 4**  Bias due to deviations from intended interventions | No information | **Comment:**  There is no information about deviations from the intended intervention. |
| **Domain 5**  Bias due to missing data | No information | **Comment:**  No information e.g. N numbers were not provided in the results tables. |
| **Domain 6**  Bias in measurements of outcomes | No information | **Comment:**  No information |
| **Domain 7**  Bias in selection of reported results | No information | **Comment:**  There is no obvious bias in the reporting of results, however there is too little information to make a judgement. There is no evidence that a pre-registered protocol or a priori statistical analysis plan existed. |
| **Overall risk of bias** | Serious | **Comment:**  There was not enough information to assess a number of domains, however the serious risk of bias rating in the confounding domain led to the same rating being applied to the overall risk of bias rating. |

**Risk of Bias Table for Barber (1988) Experiment 2**

| **Bias Domain** | **Bias judgement** | **Support for judgement** |
| --- | --- | --- |
| **Domain 1**  Bias due to confounding | Serious | **Comments:**  Barber acknowledges differences between groups but no measures to control for this were undertaken.  **Quote:**  First, the experimental procedure precluded random assignment to trained and untrained conditions. Instead, a group of already trained students was compared with a group of new arrivals at the university. It is quite probable, therefore, that the two groups differed in ways other than level of casework training. We cannot conclude unequivocally that microskills training alone is responsible for the differences reported. It may be due to a range of influences associated with the transition from first year to fourth year. Results may even have been influenced by differences in the kinds of individuals recruited to the two groups. Such explanations can never be ruled out when the experimental procedure entails non-random assignment to one or more conditions’ (p. 8)  **Comment:**  Attempts to control for confounding were insufficient, producing serious bias rating for experiment 2. |
| **Domain 2**  Bias in selection of participants into the studies | Low | **Comment:**  The information about sampling does not suggest that students were selected on the basis of participant characteristics observed after the start of the intervention. |
| **Domain 3**  Bias in classification of interventions | Low | **Comment:**  Bias in the classification of interventions is unlikely when the intervention occurs at population level. |
| **Domain 4**  Bias due to deviations from intended interventions | No information | **Comment:**  There is no information about deviations from the intended intervention. |
| **Domain 5**  Bias due to missing data | No information | **Comment:**  No information is reported about missing data or the potential for data to be missing. |
| **Domain 6**  Bias in measurements of outcomes | No information | **Comment:**  No information. |
| **Domain 7**  Bias in selection of reported results | No information | **Comment:**  There is no obvious bias in the reporting of results, however there is too little information to make a judgement. There is no evidence that a pre-registered protocol or a priori statistical analysis plan existed. |
| **Overall risk of bias** | Serious | **Comment:**  There was not enough information to assess a number of domains, however the serious risk of bias rating in the confounding domain led to the same rating being applied to the overall risk of bias rating. |

**Risk of Bias Table for Collins (1984)**

| **Bias Domain** | **Bias judgement** | **Support for judgement** |
| --- | --- | --- |
| **Domain 1**  Bias due to confounding | Moderate | **Quote:**  ‘The sample group was self-selected from the entire population of full-time first year students and, therefore, is not random’ (p. 67).  **Comment:**  Collins (1984) measured pre-existing group differences, analysed them using a chi-squared test and found them to be unproblematic |
| **Domain 2**  Bias in selection of participants into the studies | Low | **Comment:**  The information about sampling does not suggest that students were selected on the basis of participant characteristics observed after the start of the intervention. |
| **Domain 3**  Bias in classification of interventions | Serious | **Quote:**  ‘It was not possible to establish a control group where no laboratory training took place’ (Collins, 1984, p. 67)  **Comment:**  This suggests the lecture-trained and lab-trained groups were not distinctly different – the risk of bias here is serious. |
| **Domain 4**  Bias due to deviations from intended interventions | No information | **Comment:**  There is no information about deviations from the intended intervention. |
| **Domain 5**  Bias due to missing data | Low | **Comment:**  No evidence of missing data. |
| **Domain 6**  Bias in measurements of outcomes | Moderate  (analogue measure) | **Quote:**  ‘The first (pre-training) analogue was not completed until after 3 weeks of skill training had actually occurred’ (p. 98).  ‘Confounding variable are the different time periods the measures were taken’ (p. 126).  **Comment:**  There was a delay of approximately 3 weeks occurring in students completing the analogue measures, which reduced the time gap between pre-and-post-test training scores. This could have led to an under-estimation of the positive gains made by students on this outcome measure. |
|  | Low  (for other measures) | **Comment:**  Outcome assessors were not aware of which group the student belonged to, whether the clients were real or analogue or whether test measures were pre or post training. |
| **Domain 7**  Bias in selection of reported results | No information | **Comment:**  There is no obvious bias in the reporting of results, however there is too little information to make a judgement. There is no evidence that a pre-registered protocol or a priori statistical analysis plan existed. |
| **Overall risk of bias** | Serious | **Comment:**  At least one domain was assessed as a serious risk of bias, which means the overall bias rating was also rated serious. |

**Risk of Bias Table for Keefe (1979)**

| **Bias Domain** | **Bias judgement** | **Support for judgement** |
| --- | --- | --- |
| **Domain 1**  Bias due to confounding | No information | **Quote:**  ‘Three samples were drawn at random, using a table of random numbers, from second-year social work master’s students at the University of Utah School of Social Work’ (p.34).  **Comment:**  However, there is no information about the number of students in the sampling frame |
| **Domain 2**  Bias in selection of participants into the studies | Low | **Comment:**  The information about sampling does not suggest that students were selected on the basis of participant characteristics observed after the start of the intervention. |
| **Domain 3**  Bias in classification of interventions | Low | **Comment:**  Bias in the classification of interventions is unlikely when the intervention occurs at population level. |
| **Domain 4**  Bias due to deviations from intended interventions | No information | **Comment:**  There is no information about deviations from the intended intervention. |
| **Domain 5**  Bias due to missing data | Low | **Comment:**  No evidence of missing data. |
| **Domain 6**  Bias in measurements of outcomes | Serious | **Comment:**  The students were tested after their respective interventions, but the interventions were not of the same duration.  **Quote:**  ‘The groups were all pretested on the Affective Sensitivity Scale. The experimental groups were tested again following the therapeutic communications training and meditation training respectively. The control group was tested following a period of time corresponding to the length of the Therapeutic Communications course. The meditation group was tested a third time following exposure to the Therapeutic Communications course to provide an index of the effects of the two conditions combined’ (p. 34). |
| **Domain 7**  Bias in selection of reported results | No information | **Comment:**  There is no obvious bias in the reporting of results, however there is too little information to make a judgement. There is no evidence that a pre-registered protocol or a priori statistical analysis plan existed. |
| **Overall risk of bias** | Serious | **Comment:**  At least one domain was assessed as serious risk of bias, which meant the overall bias rating was also serious. |

**Risk of Bias Table for Ouellette et al. (2006)**

| **Bias Domain** | **Bias judgement** | **Support for judgement** |
| --- | --- | --- |
| **Domain 1**  Bias due to confounding | Moderate | **Quote:**  Prior to analyzing differences between the two groups, we examined the following possible confounding variables to determine if there were group differences; age, credit hours taken during the semester, previous interviewing experience, grade point average, and hours per week of paid employment during the semester. Age was the only variable found to be statistically significant. In addition, the online group was found to be slightly older than the classroom group. To determine if age was a factor in learning, a Kendall’s tau_b correlation was computed between age and all the variables in the interviewing rating scale. Only one item was found to be significant to age, that of summary statements at the end of the interviewing sequence (pp. 63-64). |
| **Domain 2**  Bias in selection of participants into the studies | Low | **Comment:**  The information about sampling does not suggest that students were selected on the basis of participant characteristics observed after the start of the intervention.  **Quote:**  Both courses occurred during the same semester and were implemented during the same time frame (p. 58). |
| **Domain 3**  Bias in classification of interventions | Low | **Comment:**  Bias in the classification of interventions is unlikely when the intervention occurs at population level. |
| **Domain 4**  Bias due to deviations from intended interventions | No information | **Comment:**  There is no information about deviations from the intended intervention. |
| **Domain 5**  Bias due to missing data | Low | **Comment:**  Of the 30 students who agreed to volunteer in the video tape interviews, only one did not participate  **Quote:**  The one student that did not, successfully completed the course but was not was not available to conduct the simulated interview at the end of the course (p. 59).  ‘A 21-item instrument was developed for use by the rater to assess the 29 interviews that were actually completed’ (p. 61).. |
| **Domain 6**  Bias in measurements of outcomes | Low | **Quote:**  ‘All videotaped interviews conducted by the students for the purpose of this study were evaluated by an independent evaluator and seasoned practitioner and social work educator with extensive clinical experience and expertise on social work interviewing skills. The independent evaluator was not aware of the group (i.e., classroom vs. on-line) to which participants belonged’ (p. 69). |
| **Domain 7**  Bias in selection of reported results | No information | **Comment:**  There is no obvious bias in the reporting of results, however there is too little information to make a judgement. There is no evidence that a pre-registered protocol or a priori statistical analysis plan existed. |
| **Overall risk of bias** | Moderate | **Comment:**  At least one domain was assessed as moderate risk, which means the overall bias rating was also moderate risk. |

**Risk of Bias Table for Rawlings (2008)**

| **Bias Domain** | **Bias judgement** | **Support for judgement** |
| --- | --- | --- |
| **Domain 1**  Bias due to confounding | Moderate | **Quote:**  ‘A convenience sample from a single institution was recruited to establish two groups, entering BSW students and exiting BSW students’ (p. 76).  **Quote:**  ‘Thirty-two students participated in the study. Sixteen students from the Senior Seminar class, 71% of the students enrolled, and 16 students from the Introduction to Social Work course, an approximate 27% of students in the course. Sampling from three offerings of the Introduction to Social Work course over three semesters was necessary to get an adequate sample.  **Comment:**  Sought to establish comparability of groups based on sex, ethnicity, grade point average, and age. |
| **Domain 2**  Bias in selection of participants into the studies | Low | **Comment:**  The information about sampling does not suggest that students were selected on the basis of participant characteristics observed after the start of the intervention. |
| **Domain 3**  Bias in classification of interventions | Low | **Comment:**  Bias in the classification of interventions is unlikely when the intervention occurs at population level. |
| **Domain 4**  Bias due to deviations from intended interventions | No information | **Comment:**  There is no information about deviations from the intended intervention. |
| **Domain 5**  Bias due to missing data | Serious | **Comment:**  Results were reported as if all student data were present, however the results tables do not provide the N numbers, and, as shown by the comments below, data were missing for some students. Missing data occurs for entering students only. It is a concern that the results tables do not acknowledge or explain the missing data.  **Quote:**  ‘Five entering BSW subjects who did not enter a GPA’ (p.75).  (This is not reflected in the results for table 4.2, p.78)  However, GPA was dropped as a control due to high numbers of missing data for the entry level group (p. 69).  **Quote:**  ‘Due to technical problems with sound, six entering students who had completed interviews were dropped from the study’ (p. 60).  But in the results graph (Figures 4.2 and 4.3) for the direct practice outcomes, all 16 students are included  **Comment:**  No attempts to use imputational approaches were used in this study; missingness was not explained. |
| **Domain 6**  Bias in measurements of outcomes | Low  (direct practice) | **Quote:**  After all of the interviews were completed, the videotapes were randomly assigned to independent raters for review using a random table of numbers. Raters were paid $15 for each tape reviewed. Five tapes were randomly selected to be assessed by both raters in order to test for inter-rater reliability. Raters were blind to the course standing of the students (p. 59).  **Quote:**  ‘Student performance in conducting an initial assessment was directly observed and evaluated by independent raters’ (p.98). |
|  | Moderate  (self-efficacy) | **Comment:**  Although the self-efficacy scales are subjective measures whereby students constitute the outcomes assessors, the way in which these were completed are unlikely to be influenced by knowledge of the intervention status. This is because the students were distinct groups of entering and exiting students (comprising entirely separate cohorts).  **Note:** The moderate rating might be generous but is in keeping with how reviewers rated the self-assessment instruments of the randomised studies. |
| **Domain 7**  Bias in selection of reported results | No information | **Comment:**  There is no obvious bias in the reporting of results, however there is too little information to make a judgement. There is no evidence that a pre-registered protocol or a priori statistical analysis plan existed. |
| **Overall risk of bias** | Serious | **Comment:**  At least one domain was assessed as having a serious risk, which means the overall bias rating was also at serious risk of bias. |

**Risk of Bias Table for Toseland and Spielberg, 1982**

| **Bias Domain** | **Bias judgement** | **Support for judgement** |
| --- | --- | --- |
| **Domain 1**  Bias due to confounding | Moderate | **Quote:**  **‘**In order to assure that students in the skills development classes were similar to students in the control group class, personal and background characteristics - such as age, previous education, marital status, and previous human service job experience - were obtained on both groups’ (p. 69).  **Quote:**  Chi-square and Kendall’s Tau were used to compare treatment and control group students. None of the differences between treatment and control group students was statistically significant, indicating that students in each group were similar to one another regarding these characteristics (p. 69). |
| **Domain 2**  Bias in selection of participants into the studies | Low | **Comment:**  The information about sampling does not suggest that students were selected on the basis of participant characteristics observed after the start of the intervention. |
| **Domain 3**  Bias in classification of interventions | Low | **Comment:**  Bias in the classification of interventions is unlikely when the intervention occurs at population level. |
| **Domain 4**  Bias due to deviations from intended interventions | No information | **Comment:**  There is no information about deviations from the intended intervention. |
| **Domain 5**  Bias due to missing data | Low | **Comment:**  No information is reported about missing data or the potential for data to be missing. N numbers of students are included in the results table, suggesting attrition did not occur. |
| **Domain 6**  Bias in measurements of outcomes | No information | **Comment:**  There is no information regarding whether outcome assessors knew which group the students belonged to. |
| **Domain 7**  Bias in selection of reported results | No information | **Comment:**  There is no obvious bias in the reporting of results, however there is too little information to make a judgement. There is no evidence that a pre-registered protocol or a priori statistical analysis plan existed. |
| **Overall risk of bias** | Moderate | **Comment:**  At least one domain was assessed as having a moderate risk of bias, which means the overall bias rating was also a moderate risk of bias. |

**Risk of Bias Table for VanCleave, 2007**

| **Bias Domain** | **Bias judgement** | **Support for judgement** |
| --- | --- | --- |
| **Domain 1**  Bias due to confounding | Moderate | **Quote:**  Participants were recruited as a convenience sample from the eight master’s level social work cohorts (p. 106).  **Quote:**  Approximately one half of the students agreeing to participate were placed in the intervention group. The remaining half was part of the comparison group (p. 105).  **Quote:**  ‘Selection of a non-random design subjected the research to confounds and threats to validity’ (VanCleave, 2007, p. 105).  **Quote:**  ‘This research has considered variables which might present confounding influence’ (p.32).  **Quote:**  ‘No attempt was made to separate the groups based upon years of experience in the field, or based upon age. However, distributions fell pretty evenly…’ (p.135). |
| **Domain 2**  Bias in selection of participants into the studies | Low | **Comment:**  The information about sampling does not suggest that students were selected on the basis of participant characteristics observed after the start of the intervention. |
| **Domain 3**  Bias in classification of interventions | Low | **Comment:**  Bias in the classification of interventions is unlikely when the intervention occurs at population level. |
| **Domain 4**  Bias due to deviations from intended interventions | No information | **Comment:**  There is no information about deviations from the intended intervention. |
| **Domain 5**  Bias due to missing data | Low | **Comment:**  The students who dropped out were accounted for and the reasons for drop out were stated, where they had been provided.  **Quote:**  Of the five volunteers who did not participate, three came from the training group and two came from the control (p. 156). |
| **Domain 6**  Bias in measurements of outcomes | Serious  (Empathic concern) | **Comment:**  The researcher was affiliated to the university where the research was conducted.  **Quote:**  The training was conducted by the researcher, who has nineteen years of teaching as an adjunct in the university where the training occurred (p.25).  **Quote:**  The researcher acted as teacher and facilitator in the intervention, which is typically not a recommended research strategy. The researcher had the required experience, expertise, and motivation to complete the intervention; however, supervisory faculty at USI oversaw the intervention to assist in intervention and program integrity (p. 117).  **Comment:**  The Davis IRI self-inventory is a self-assessment instrument whereby students rated their own empathic concern and perspective taking.  **Quote:**  ‘The Davis self-inventory was completed by the participant before, or following, each 8 excerpt role played situation’ (VanCleave, 2007, p.118).  **Comment:**  Inconsistency surrounding the timing of when the instrument was completed led to a serious bias rating for the outcome measure of empathic concern and perspective taking. |
|  | Low  (Empathic response) | **Quote:**  The participant student, in the intervention group, began the project by audiovisual taping the 8 role plays prior to the empathy training, as a pretest. Posttesting for the intervention and control group was set at the conclusion of the training workshop (p.111).  **Quote:**  The expert raters evaluated the audiovisual tape of each of the test role plays, from both groups (p. 113).  **Quote:**  Both pretaping and posttaping VHS cassettes were coded, for the primary researcher’s identification only, as an intervention or a control tape.  **Comment:**  Only the primary researcher (not the expert raters) knew whether the videotapes they were assessing belonged to a student in the intervention or the control group. |
| **Domain 7**  Bias in selection of reported results | No information | **Comment:**  There is no obvious bias in the reporting of results, however there is too little information to make a judgement. There is no evidence that a pre-registered protocol or a priori statistical analysis plan existed. |
| **Overall risk of bias** | Serious  (Empathic concern) | **Comment:**  At least one outcome measure was assessed as serious risk of bias, which means the overall bias rating was also serious. |
|  | Moderate (Empathic response) | **Comment:**  At least one domain was assessed as moderate risk of bias, which means the overall bias rating was also moderate. |

**Risk of Bias Table for Vinton and Harrington, 1994**

| **Bias Domain** | **Bias judgement** | **Support for judgement** |
| --- | --- | --- |
| **Domain 1**  Bias due to confounding | No information | **Comment:**  Lack of information from which to judge bias rating. |
| **Domain 2**  Bias in selection of participants into the studies | Low | **Comment:**  The information about sampling does not suggest that students were selected on the basis of participant characteristics observed after the start of the intervention. |
| **Domain 3**  Bias in classification of interventions | Low | **Comment:**  Bias in the classification of interventions is unlikely when the intervention occurs at population level. |
| **Domain 4**  Bias due to deviations from intended interventions | No information | **Comment:**  There is no information about deviations from the intended intervention. |
| **Domain 5**  Bias due to missing data | Low  (QMEE) | **Comment:**  There does not appear to be any missing data for the QMEE scores, as results for all 62 students are accounted for (table 1). |
|  | No information (Carkhuff scales) | **Comment:**  For the Carkhuff scale scores, a judgement cannot be made because the N numbers are not provided in the results table (table 2). |
| **Domain 6**  Bias in measurements of outcomes | Serious  (Emotional empathy - QMEE) | **Comment:**  Students rated their own empathy on the QMEE.  **Quote:**  ‘Neither of the experimental groups was seen to significantly improve in terms of QMEE scores. Furthermore, the control group scores were actually seen to significantly decrease at posttest’ (p. 79).  **Comment:**  Potentially the control group might have scored lower because they were aware they were not receiving the same interventions as their peers |
|  | No information (Expressed empathy -  Carkhuff scales) | **Comment:**  Two raters assessed the students’ levels of empathy using the Carkhuff scale. There is no information about whether the raters were independent or blinded to the intervention status of the students they assessed. Researcher allegiance could potentially be a problem but there is no information on which to make this judgement. |
| **Domain 7**  Bias in selection of reported results | No information | **Comment:**  There is no obvious bias in the reporting of results, however there is too little information to make a judgement. There is no evidence that a pre-registered protocol or a priori statistical analysis plan existed. |
| **Overall risk of bias** | Serious  (Emotional empathy - QMEE) | At least one domain was assessed as high risk of bias, which means the overall bias rating was also high. |
|  | No information  (Expressed empathy -  Carkhuff scales) | **Comment:**  The lack of information in almost all of the domains, meant there was not enough information to provide an overall bias rating for this study. |
